# On-demand storage and release of antimicrobial peptides using Pandora's box-like nanotubes gated with a bacterial infection-responsive polymer

**DOI:** 10.7150/thno.38388

**Published:** 2020-01-01

**Authors:** Junjian Chen, Xuetao Shi, Ye Zhu, Yunhua Chen, Meng Gao, Huichang Gao, Lei Liu, Lin Wang, Chuanbin Mao, Yingjun Wang

**Affiliations:** 1 National Engineering Research Center for Tissue Restoration and Reconstruction, South China University of Technology, Guangzhou 510006, China.; 2 Key Laboratory of Biomedical Materials and Engineering of the Ministry of Education, South China University of Technology, Guangzhou 510641, China.; 3 Department of Chemistry and Biochemistry, University of Oklahoma, Stephenson Life Sciences Research Center Norman, OK, 73019, USA.; 4 School of Biomedical Science and Engineering, South China University of Technology, Guangzhou 510006, China.; 5 Key Laboratory of Biomedical Engineering of Guangdong Province, South China University of Technology, Guangzhou 510006, China.; 6 Guangzhou Regenerative Medicine and Health Guangdong Laboratory, Guangzhou 510006, China.

**Keywords:** Titania nanotubes, pH-Responsive molecular gate, On-demand delivery, Bactericidal activity, Peptides

## Abstract

**Background**: Localized delivery of antimicrobial agents such as antimicrobial peptides (AMPs) by a biomaterial should be on-demand. Namely, AMPs should be latent and biocompatible in the absence of bacterial infection, but released in an amount enough to kill bacteria immediately in response to bacterial infection.

**Methods**: To achieve the unmet goal of such on-demand delivery, here we turned a titanium implant with titania nanotubes (Ti-NTs) into a Pandora's box. The box was loaded with AMPs (HHC36 peptides, with a sequence of KRWWKWWRR) inside the nanotubes and “closed” (surface-modified) with a pH-responsive molecular gate, poly(methacrylic acid) (PMAA), which swelled under normal physiological conditions (pH 7.4) but collapsed under bacterial infection (pH ≤ 6.0). Thus, the PMAA-gated Ti-NTs behaved just like a Pandora's box. The box retarded the burst release of AMPs under physiological conditions because the gate swelled to block the nanotubes opening. However, it was opened to release AMPs to kill bacteria immediately when bacterial infection occurred to lowering the pH (and thus made the gate collapse).

**Results**: We demonstrated such smart excellent bactericidal activity against a panel of four clinically important bacteria, including *Staphylococcus aureus*, *Escherichia coli*, *Pseudomonas aeruginosa*, methicillin-resistant *Staphylococcus aureus*. In addition, this box was biocompatible and could promote the osteogenic differentiation of human mesenchymal stem cells. Both *in vitro* and *in vivo* studies confirmed the smart “on-demand” bactericidal activity of the Pandora's box. The molecularly gated Pandora's box design represents a new strategy in smart drug delivery.

## Introduction

Biomaterial-associated infection (BAI) remains a serious global problem, especially for orthopaedic titanium implants 1-6. This infection may occur after surgery, especially at initial stage (acute infection stage) 7, 8. At this stage, pathogens are inclined to aggregate on the implants, and form bacterial biofilms to protect themselves against host immune defences and environmental stresses 9. The BAI has serious consequences, including the failure of surgeries, disability, sepsis and even death of patients 10-12. In the clinic, besides cleaned the suture of wound with 75% ethanol, systemic administration of antibiotics must be widely applied after surgery to inhibit bacterial infection. However, this method displays low efficiency, cytotoxicity and drug resistance 13-15. Thus localized release of antimicrobial agents from implants was proposed to be a more efficient antimicrobial approach 16-20. This approach can achieve an adequate concentration of antimicrobial agents at the target site to combat bacteria 21-24, and can also display negligible systemic impact to reduce the cytotoxicity to the patients 7. Meanwhile, antimicrobial peptides (AMPs) are considered to be one of the desired antimicrobial agents because of their low drug resistance and broad-spectrum bactericidal activity 25-30. Covalently immobilized AMPs show poor stability against enzymes and low activity against the untouched bacteria around the tissues 31. Thus, localized delivery of AMPs is more desired and indeed found to be much better at protecting the AMPs from degradation and killing the bacteria on/around the implant 7, 32, while displaying lower cytotoxicity than systemic administration. Currently, the localized delivery of AMPs is usually achieved by loading AMPs on a substrate, such as calcium phosphate films 32, or by the interaction between TiO_2_ and anchor sequence 33, 34. Additionally, titania nanotubes (TiO_2_-nanotubes, Ti-NTs) have attracted much attention due to their unique topography, high surface-to-volume ratio, and excellent loading capability 7, 35.

However, the reported designs of localized delivery of AMPs did not consider the different demands in cases with or without bacterial infection 7, 36, 37. AMPs release should be in an “on-demand” manner. Namely, under noninfective conditions, the release of AMPs should be slow to further reduce their cytotoxicity 35. Under infective conditions, the AMPs-modified materials should respond to the incoming bacteria and rapidly release AMPs to immediately show a strong bactericidal activity. Based on other reports, bacterial infection could produce acidic substrates, such as lactic and acetic acid 3, leading to a pH decrease to 5.0 at local infection sites, which had been employed by the researchers to develop antimicrobial/antifouling surfaces 3, 38-40 or nanotechnological loading of agents 41, 42, but was rare in the modification of localized delivery system on titanium implants, especially Ti-NTs.

**Scheme 1:**
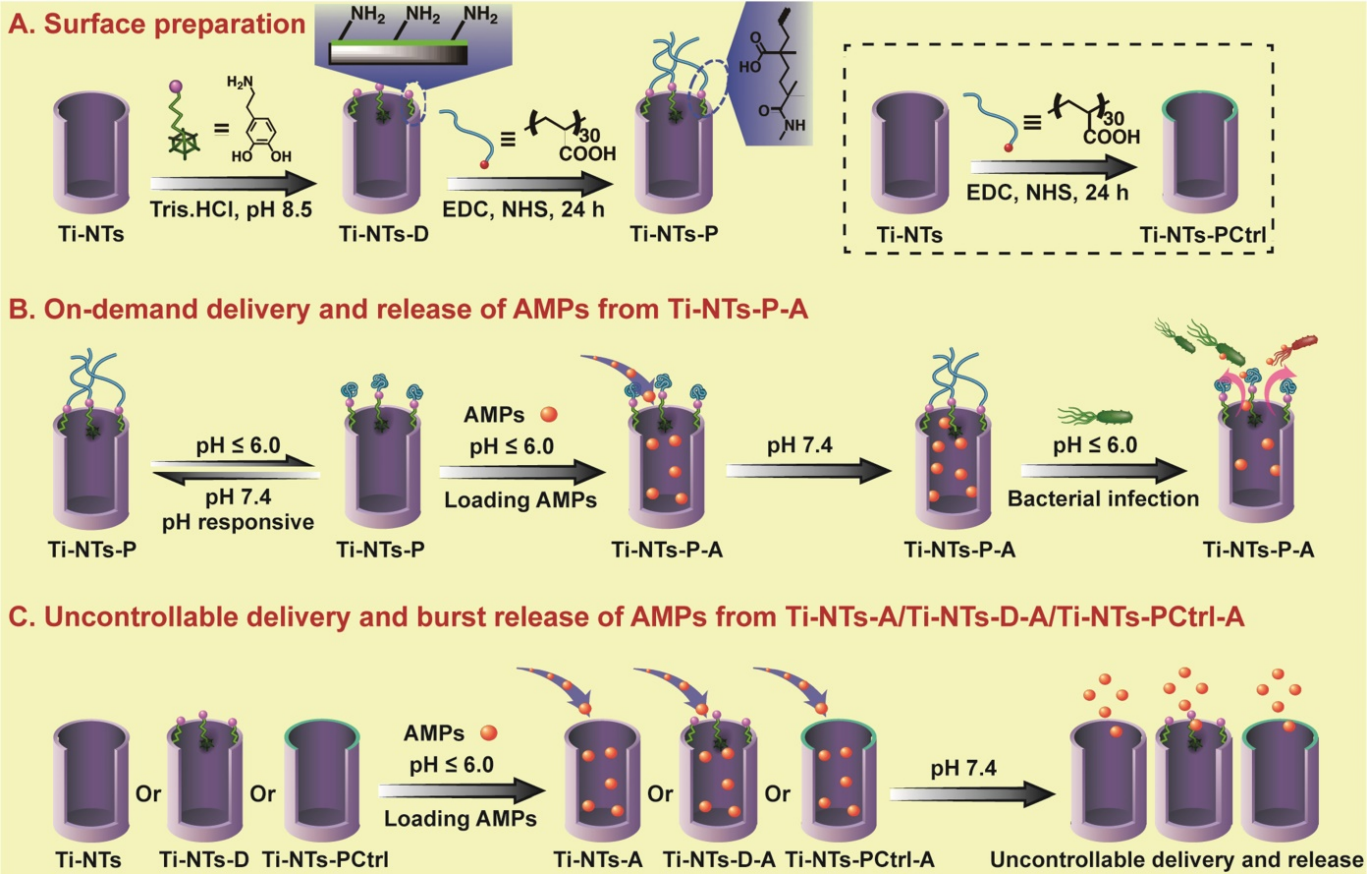
** The preparation of the Pandora's box as well as the loading and releasing of AMPs into and from the box, respectively. (A)** The preparation of the Pandora's box, the PMAA “gates” engineered Ti-NTs (Ti-NTs-P), and its control groups. First, Ti-NTs were modified with dopamine to form Ti-NTs-D. Ti-NTs-D were conjugated with the gate molecule, PMAA, to from Ti-NTs-P. Ti-NTs were also directly modified with PMAA to form a control substrate called Ti-NTs-PCtrl. **(B)** The loading and “on-demand” delivery of AMPs into and out of the Pandora's box. First, to load PMAA, the box was placed in an AMPs solution with a pH value of less than 6. The low pH made the chains of PMAA gate molecules collapsed and the nanotubes opened, enabling the loading of AMPs into the nanotubes. Then the pH of the solution was increased to 7.4 to make the PMAA molecules swell, leading to the encapsulation of the loaded AMPs to form Ti-NTs-P-A. When Ti-NTs-P-A substrates were in an environment where bacterial infection occurred (causing the environmental pH dropped to below 6), the PMAA gate molecules will collapse to open the nanotubes, leading to the rapid release of the AMPs to immediately kill the bacteria. Otherwise, the AMPs in the nanotubes will be “latent”. **(C)** The loading and uncontrollable delivery of AMPs on control substrates due to the absence of the gate PMAA molecules, including Ti-NTs, Ti-NTs-D and Ti-NTs-PCtrl. EDC: 1-ethyl-3-(3-dimethylaminopropyl)-carbodiimide hydrochloride; NHS, N-hydroxysuccinimide; PMAA, polymethacrylic acid.

Specifically, in the present study, we first immobilized dopamine onto Ti-NTs to form dopamine-modified surface (termed Ti-NTs-D) as in the reference 43. As one of the effective pH-responsive polymers, poly(methacrylic acid) (PMAA) was covalently conjugated onto Ti-NTs-D surface via the interaction between the carboxyl group on PMAA and the amino group on dopamine to generate PMAA-modified surface (termed Ti-NTs-P) (Scheme 1A). As reported, PMAA swelled in pH ~ 7.4 (physiological environments in the absence of bacterial infection) and collapsed in pH ≤ 6.0 (acidic micro-environments generated due to bacterial infection) (Scheme 1B) 40. Thus, PMAA served as an infection-sensitive and switchable molecular “gate” to achieve sustained and on-demand release of the AMPs in response to bacterial infection like a Pandora's box (Scheme 1B). We took advantage of the box to load and release HHC36 peptides, one of the most potent broad-spectrum AMPs with an excellent bactericidal activity 7. This box was expected to achieve the ideal status that released AMPs at slow rate to prevent the latent bacterial infection and keep biocompatibility under physiological condition, and show a burst release of AMPs when bacterial infection occur.

As a proof-of-concept, the controlled release profiles of AMPs were comprehensively studied at different pH values. We confirmed the bactericidal activities of this system against four clinically relevant bacteria, i.e. *Staphylococcus aureus* (*S. aureus*), *Escherichia coli* (*E. coli*), *Pseudomonas aeruginosa* (*P. aeruginosa*), methicillin-resistant *Staphylococcus aureus* (*MRSA*), as well as cytotoxicity/osteogenic activity for *human bone mesenchymal stem cells* (*hBMSCs*). Moreover, *in vivo* bactericidal activity and biocompatibility were also verified in a bone defect model of New Zealand rabbits.

## Materials and Methods

**Materials:** Cells (*Human bone mesenchymal stem cells*, *hBMSCs*, Catalog #7500, ScienCell Research Laboratories, California, USA), Bacteria (*S. aureus* (ATCC 29213), *E. coli* (ATCC 8739), *P. aeruginosa* (ATCC 15442) and *MASA* (ATCC 43300), VWR International, LLC, Pennsylvania, USA), HHC36 peptides (95%, without further modification, China Peptides Co., Ltd., Shanghai, China), titanium (size: 1 cm × 1 cm × 0.1 mm or Φ 2 mm × 3 mm, Chenhui Metal Materials Ltd., Shanxi, China), the CCK-8 kit (Dojindo, Kumamoto, Japan), polymethacrylic acid (PMAA, Mw≈9500, Sigma-Aldrich, Missouri, USA), doxycycline hyclate (Aladdin, Shanghai, China) were purchased.

**Surface modification:** Ti substrates (including wafers and rods) were treated with aqueous solution containing 3 vol% HF for 1 min and were used as an anode, while platinum foil was used as a cathode. Both the anode and cathode were immersed in electrolyte solution (200 mL) containing NH_4_F (0.5 wt%) and (NH_4_)_2_SO_4_ (1 M) at a voltage of 20 V for 30 min with the distance of 5 cm. After anodization and washing, the anode was heated to 500 °C (5 °C/min), held for 3 h, and cooled. The obtained sample was denoted Ti-NTs (Table 1).

Ti-NTs were treated with oxygen plasma for 5 min and incubated in 1 mL of 3,4-dihydroxyphenethylamine hydrochloride (dopamine) in Tris-buffer (1 mg/mL, pH was adjusted to 8.5 by 1 M NaOH). After 24 h, the dopamine-treated Ti-NTs were washed and denoted Ti-NTs-D (Table 1). To immobilize PMAA molecules onto the nanotubes, Ti-NTs-D was immersed in a mixed solution having 1-ethyl-3-(3-dimethylaminopropyl)-carbodiimide hydrochloride (EDC, 50 mM), N-hydroxysuccinimide (NHS, 20 mM) and 5 mM PMAA. After 24 h, the sample was washed and denoted Ti-NTs-P (Table 1). Additionally, we prepared the control group by directly immersing Ti-NTs (without dopamine) into PMAA solution under the same procedure and denoted it Ti-NTs-PCtrl (Table 1).

**Surface characterization:** Scanning electron microscope (SEM, Quanta 200, FEI), X-ray photoelectron spectroscopy (XPS, Kratos Axis Ultra), contact angle (OCA15 contact angle goniometer, Dataphysics, Germany), X-ray diffraction (XRD, X'Pert Pro, PANalytical B.V.), ultraviolet absorption spectroscopy (UV absorption, UV-2600, Shimadzu, Japan) were performed.

**Loading and release of HHC36 peptides:** Then, 50 μL of HHC36 peptides solution (1 mM in ethanol (1500 μg/mL), and the pH was adjusted to 5.0 by HCl solution for collapsing the PMAA molecules) was added onto each substrate. The substrates were dried under vacuum desiccator, and this process was repeated 10 times. After that, the samples with HHC36 peptides were rinsed with PBS and named Ti-NTs-A, Ti-NTs-D-A, Ti-NTs-PCtrl-A and Ti-NTs-P-A, respectively (Table 1).

The samples with HHC36 peptides were placed in 24-well plates, and 250 μL of PBS was poured into each well to make the samples immersed at 37 °C. The incubation solution was collected at the indicated time points. Then, the rate of the HHC36 peptides release from different substrates was calculated by measuring the UV absorption of the collected solution at OD_280_. We also characterized the release of the peptides in different microenvironments by adjusting the pH values of the PBS with 0.1 M HCl solution.

**Bacterial culture:** The bacteria were streaked onto agar plates and cultured for 12 h. After that, individual colonies of each bacterium were picked and routinely pre-cultured in LB medium (3 mL) with shaking (220 rpm). At a mid-log phase growth period, 10 μL of bacterial suspension was re-suspended in 1 mL LB medium to a specific concentration according to the optical density (OD) values at 600 nm.

**Minimum inhibitory concentration (MIC) and minimum bactericidal concentration (MBC) assay:** Bacterial suspension and peptides solutions were mixed at a given concentration. The systems were cultured in a microtiter plate at 37 °C, and the bacterial concentration was determined by an ELISA microplate reader (Varioskan Flash 3001). The MICs were determined by subtracting the background. For MBC assay, the AMPs were added to the bacterial solution (5 × 10^5^ CFU/mL) to achieve the final concentration from 0 to 50 μM. After being cultured for 18 h, the bacterial suspension was diluted by 10^1^, 10^2^, 10^3^ and 10^4^ times with PBS, and the MBC values were determined by evaluating the viability of the bacteria in 10 μL of the suspension with agar plates as in the reference 44.

***In vitro* bactericidal assay:** 250 μL of bacteria suspension, diluted in LB medium (1 × 10^7^ CFU/mL), was added to fully cover the surface. After 1 h in culture, the bacterial suspension was immediately transferred from the 24-well plate to Eppendorf tubes and each suspension was taken to streak onto agar plates. The amount of bacteria was determined after 15 h. The substrates were air-dried, and the above procedures were repeated 3 more times.

For the bactericidal activity in 7 days, the substrates were immersed in 250 μL of PBS solution at 37 °C for different durations (1-7 days). After the substrates were rinsed by distilled water, 250 μL of the suspensions (with the bacterial concentration of 1 × 10^7^ CFU/mL) was added and cultured for the indicated times. Herein, we estimated the culture time according to the amount of released AMPs. We calculated the quantity of AMPs released from Ti-NTs-P-A in a physiological environment in the first 1 h (Q_1h_). Then, the culturing times were estimated by a release curve constructed from experiments in the physiological environment, where the amount of released AMPs was the same as Q_1h_. It should be noted that the estimated culturing times were deviated from those of the release curve in the physiological environment, as the bacteria would alter the pH values and the release rate. To evaluate Ti-NTs-P-A's sustained bactericidal property compared to control groups, we believed that the deviation of the indicated time would not impact the conclusion. In the present study, the culturing times for 1 to 7 days were 4.2, 5.6, 5.8, 6.4, 6.8, 6.8 and 8.0 h, respectively. After culturing, the suspension was collected to evaluate the bactericidal property as above.

We also investigated the bactericidal activities of AMPs and the samples against *S. aureus* after steam sterilization (HVA-110, HIRAYAMA, Japan). For AMPs, after sterilization, the AMPs were added to the bacterial suspension (1 × 10^7^ CFU/mL) to achieve a final concentration of 0 to 20 μM. After cultured for 2 h, the bacterial suspension was diluted by 10^0^, 10^1^, 10^2^, 10^3^ and 10^4^ times with PBS, and 10 μL of each solution were taken for spinning on agar plates. The number of colonies on each agar plate was counted after 15 h. For the sterilized samples, we characterized their bactericidal activities as above with the bacterial suspension (1 × 10^7^ CFU/mL) by the agar plate method.

**Cell culture:** *hBMSCs* were cultured with basal medium containing fetal bovine serum (10%), L-glutamine (1%) and penicillin/streptomycin (1%) at 37 ℃ and with 5% CO_2_ (HERAcell 240i, ThermoFisher Scientific Inc., USA), and 5^th^-6^th^ passaged cells were used.

***In vitro* CCK-8 assay:** Substrates were treated with ethanol (75 vol%) for sterilization. Subsequently, 1 mL of cell suspension containing 2 × 10^4^ *hBMSCs* was added, and the substrates were incubated at 37 °C. After culturing for 1, 3 and 7 days, the samples were incubated in 350 μL of CCK-8 medium (the ratio between medium and CCK-8 solution was 10:1 in volume) for 3 h, and the optical density (OD_450 nm_) of 100 μL of the solution was characterized by the plate reader. Three replicates were used for each group to obtain the mean value.

***In vitro* vinculin staining:** After 24 h in culture of *hBMSCs*, the substrates were fixed with formaldehyde (350 μL, 4 vol%) overnight (4 °C). Then, the substrates were incubated in Triton X-100 (0.1%, 10 min) and bovine serum albumin solution (BSA, 3%, 1 h). The substrates were further treated with polyclonal rabbit anti-vinculin antibody (primary antibody) overnight (4 °C), and immersed in the secondary antibody (goat anti-rabbit IgG H&L pre-absorbed ab150087). After 1 h of culturing, the substrates were immersed in F-actin (1 h) and DAPI (4,6-diamidino-2-phenylindole, 5 min), respectively. The images of cells were performed via the confocal microscopy (Leica TCS SP5, Germany).

**Quantitative reverse transcription polymerase chain reaction (qRT-PCR):** The main differentiation markers such as alkaline phosphatase (ALP), runt-related transcription factor 2 (RUNX-2), collagen type 1 (COL1) and osteopontin (OPN) were analyzed with the housekeeping gene of GAPDH on the 7^th^ and 14^th^ days. As above, the samples were sterilized and *hBMSCs* were seeded at a density of 5 × 10^4^ per sample in osteogenic α-MEM. The ribonucleic acid (RNA) was collected with the Total RNA Kit (Magentec, China). The RNA concentrations were characterized by the NanoDrop instrument (Thermo Scientific, Finland), and the reverse transcription reactions were acquired with 500 ng of total RNA (PrimeScript RT reagent kit with gDNA Eraser, Takara Biotechnology). Consequently, we performed the qRT-PCR with the SYBR System (GeneCopoeia) by QuantStudio 6 Flex (Life Technologies) with the Primer sequences in Table S2. We obtained the relative gene quantity by normalizing to GAPDH, and the results were calculated by 2^-ΔΔCt^ method, where Ct represents the cycle number when an arbitrarily placed threshold was reached, and ΔΔCt was calculated as follows 45:


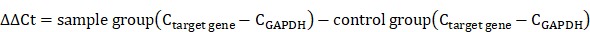


**Immunofluorescence staining:** As above, the samples were sterilized and the *hBMSCs* were seeded at a density of 5 × 10^4^ per sample in osteogenic α-MEM. After the indicated times (7 d for ALP and RUNX-2, and 14 d for OPN), the substrates were fixed by neutral formaldehyde (4%) overnight, and permeabilized by Triton X (0.1%, 10 min), blocked by BSA solution (1%, 60 min), and immersed in primary antibody ALP ((C-8): sc-373737), RUNX-2 ((27-K): sc-101145) or OPN ((LFMb-14): sc-73631) for 12 h (4 °C). After that, the substrates were immersed in secondary antibody (donkey anti-mouse IgG H&L, 1 h), F-actin (1 h) and DAPI (5 min), and cleaned by PBS. Finally, the substrates were characterized using the confocal microscopy.

**Western blot analysis:** As above, the samples were prepared and the *hBMSCs* were seeded at a density of 5 × 10^4^ per sample in osteogenic α-MEM. After being cultured for 7 and 14 days, *hBMSCs* were collected by centrifugation (12000 rpm, 4 °C, 15 min). The cytosolic proteins of *hBMSCs* were extracted with radio immune precipitation assay (RIPA) lysis buffer with a cocktail of protease and phosphatase inhibitor. Then the total extracted protein concentration was calculated using BCA Protein Assay Kit. Then, the samples were incubated in the primary antibodies as follows: anti-ALP (1:1000, DF12525, Affinity, USA), anti-RUNX-2 (1:1000, AF5186, Affinity, USA), anti-collagen I (1:750, AF7001, Affinity, USA), and anti-osteopontin (1:1500, AF0227, Affinity, USA) (overnight at 4 °C). After that, the samples were cleaned by Tris-buffered saline with 0.1% Tween-20, and incubated in the secondary antibody (anti-rabbit secondary antibody, CST, USA) for 1 h with blocking buffer with shaking. Finally, the immunoreactive bands were characterized by electro-chemiluminescence reagent (Millipore, USA).

***In vivo* assay operation:** All *in vivo* experiments were approved by the Institutional Animal Care and Use Committee of Guangdong Medical Laboratory Animal Center (Foshan, China). We employed modified rabbit osteomyelitis model for the bone infection research 46. Briefly, we anesthetized New Zealand rabbits (2.3 ± 0.15 kg, male) with 3 wt% pentobarbitalum in a dose of 0.3 mL/kg and xylazine hydrochloride injection in a dose of 0.1 mL/kg. After that, we separated the patellar ligament, drilled a hole (Φ 3 mm) on the top of the tibia, injected 40 μL of *S. aureus* solution (1 × 10^8^ CFU/mL) and implanted titanium rods. Finally, we sealed the insertion site and sutured the patellar ligament and skin.

***In vivo* antimicrobial assay:** After 7 days of implantation, the implants and tissues were extracted, incubated in LB medium, and diluted in PBS to evaluate the bacterial viability on implants. For medullary cavity assay, we froze and crushed the tibias, and ground them into powder. Then, the powder with equal quantity was added into 50 mL of LB medium and diluted in PBS to evaluate the bactericidal property.

***In vivo* H&E staining:** The tibia was treated with 4% formaldehyde (3 d), ethylenediaminetetraacetic acid (EDTA, 30 d), and ethanol gradients (50% for 120 min, 70% for 120 min, 80% for 90 min, 95% for 120 min and 100% for 90 min). The tissue was then treated with dimethylbenzene 3 times (each for 30 min), and placed in paraffin wax boxes (60 °C for 120 min). Consequently, the tissue was immersed in melting paraffin wax (63 °C), and cultured in the freezing platform. After solidified, the tissue section (slice) was acquired with a microtome, treated with dimethylbenzene 2 times (each for 15 min), 100% ethanol 3 times (each for 5 min), and cleaned by distilled water. Finally, the samples were treated with hematoxylin (2 min), eosin solution (5 min), and were dehydrated with 100% ethanol 2 times (each for 5 min) and dimethylbenzene 2 times (10 min).

Statistics: Data of all experiments were obtained from at least three independent experiments, and expressed as mean ± standard deviation (SD) by OriginPro 2018 software. We employed *SPSS* 17.0 statistical software (*t*-test) to calculate the statistical significance between different groups with *p* < 0.05 being considered statistically significant.

## Results and Discussion

### Preparation of “gates” engineered Ti-NTs substrates

**Figure 1:**
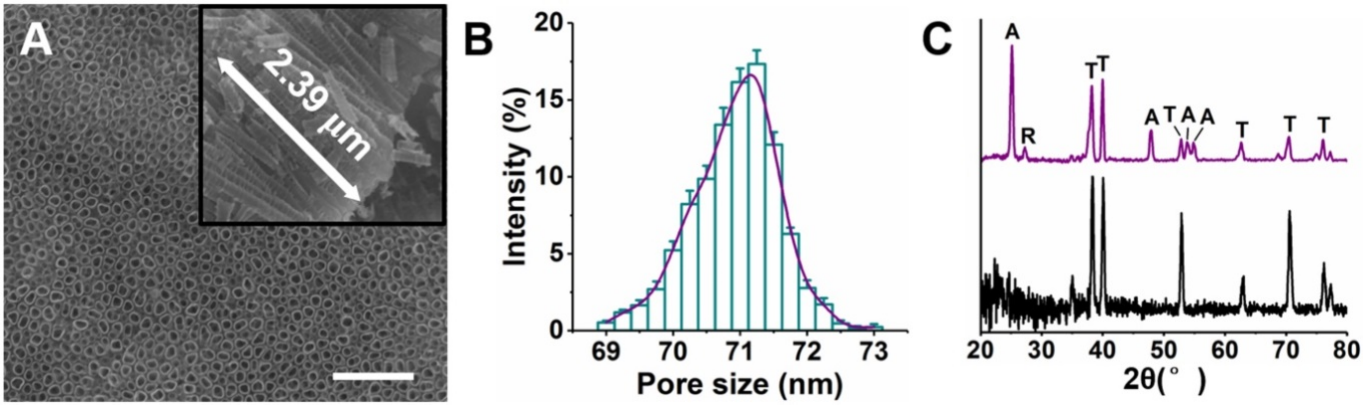
** Structures of TiO_2_ nanotubes on titanium (Ti-NTs).** (A) SEM images of the Ti-NTs. Inset: cross-section view. Scale bar, 500 nm. (B) Pore size distribution of Ti-NTs determined by Nano Measurer. Error bars denote the standard deviations over triplicate measurements with the same implants. (C) XRD pattern of Ti-NTs before (bottom) and after (top) annealing treatment. A: anatase, R: rutile, and T: amorphous.

Ti-NTs were prepared by anodic oxidation on titanium substrates. Their morphology was confirmed using scanning electron microscope (Figure 1A). The nanotubes were uniform, with an average length of approximately 2.39 μm and an average pore size of approximately 71.0 nm (Figure 1B). Moreover, the lengths of the nanotubes on Ti-NTs-D, Ti-NTs-P and Ti-NTs-A were similar to those on Ti-NTs, indicating that the grafted molecules and loaded AMPs would not affect the lengths of the nanotubes (Figure S1, SI). X-ray diffraction patterns showed that an annealing treatment enhanced the crystallinity of the substrates (a mixture of anatase and rutile phases) (Figure 1C). Such phase structure was known to promote the osteogenesis 47-49.

**Figure 2:**
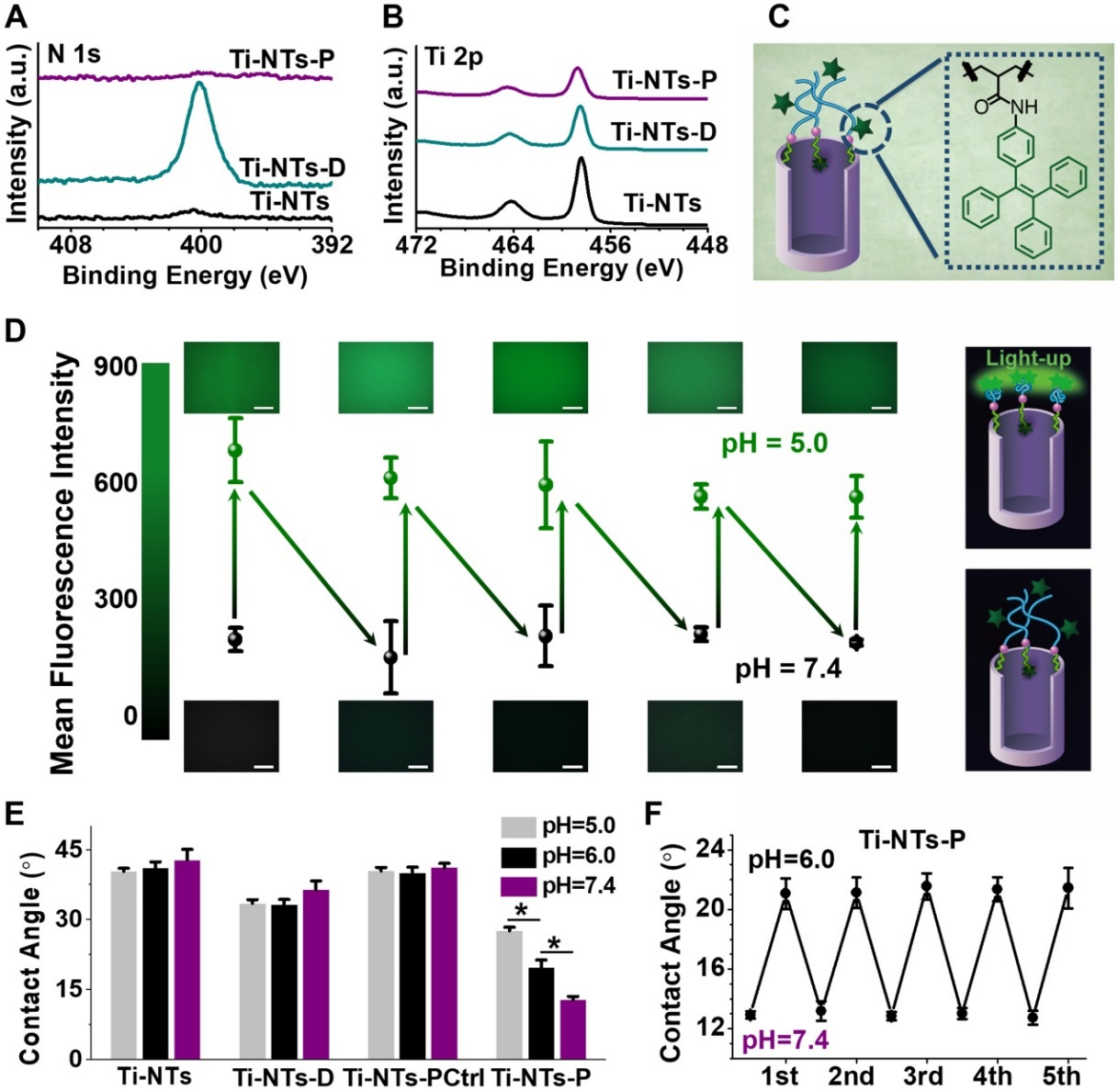
** Characterization of the “gates” engineered substrates and the pH-switchable ability of Ti-NTs-P.** (A-B) High-resolution XPS N 1s (A) and Ti 2p (B) spectra of the indicated surfaces. (C) Schematic diagram of the integration of AIEgens (TPE-NH_2_) on Ti-NTs-P. (D) Mean fluorescent intensity (MFI) of the surface with AIEgens at different pH values (5.0 ~ 7.4). Scale bar, 50 μm. (E) Water contact angles of the indicated surfaces at different pH values between 7.4 and 5.0. * denotes *p* < 0.01. (F) The contact angle variation of Ti-NTs-P with the changes in pH between 7.4 and 6.0. All error bars denote the standard deviations over triplicate measurements with separately implants.

The X-ray photoelectron spectroscopy (XPS) was employed to exhibit the grafting processes of dopamine and PMAA molecules onto the titanium implants (Figure 2A and B). After the coating of dopamine on Ti-NTs to form Ti-NTs-D, the N 1s peak appeared and the Ti 2p signal was reduced on Ti-NTs-D compared to Ti-NTs, indicating the successful immobilization of dopamine molecules. After the conjugation of PMAA with the dopamine to form Ti-NTs-P, the N 1s peak disappeared with a continuous decrease in the Ti 2p signal on Ti-NTs-P (Figure 2A and B), suggesting the successful integration of PMAA molecules. The modification could also be demonstrated by the variations in the atom ratios between elements (Table S1, SI), in which N/Ti ratio increased from 4.6% in Ti-NTs to 91.0% in Ti-NTs-D, and N/C ratio increased from 7.7% in Ti-NTs to 12.4% in Ti-NTs-D due to the grafting of dopamine molecules. Compared to their values in Ti-NTs-D, N/Ti and N/C ratio decreased to 4.0% and 1.3%, respectively, in Ti-NTs-P because of the integration of the N-deficient PMAA molecules. Meanwhile, the elemental compositions of the control surface (termed Ti-NTs-PCtrl in Scheme 1A) were similar to those of Ti-NTs; They showed a strong Ti 2p signal was observed, indicating the lack of PMAA molecules on their surface (Table S1 and Figure S2, SI). Moreover, we also employed the Acid Orange II staining 50 and UV absorption method (Figure S3, SI) to find that the grafting amount of dopamine and PMAA on Ti-NTs was 18.6 ± 7.1 nmol/cm^2^ and 4.2 ± 2.5 nmol/cm^2^, respectively.

The rapid “swelling-collapsing” transformation of the PMAA “gate” was evaluated by the aggregation-induced emission (AIE) fluorescence labelling technique. AIE luminogens (AIEgens) are fluorescent when molecularly aggregated while nonluminescent when dissolved 51-55. In the present study, we immobilized the AIEgens (TPE-NH_2_) on Ti-NTs-P by an amide coupling reaction (Figure 2C). After modification, the surface displayed weak fluorescence with a mean fluorescent intensity (MFI) of less than 300 a.u. at pH 7.4 (Figure 2D), suggesting the insufficient aggregation of AIEgens due to the swelling of the PMAA molecules. When we decreased the pH to 5.0, the fluorescence was stimulated, and the MFI increased to approximately 600 a.u., indicating that the PMAA molecules were changed from swelling to collapsing. This change was repeatable, and the MFI of the surface decreased to less than 300 a.u. again when the pH increased to 7.4. Moreover, after pH value was changed, the MFI of the surface could be stabilized within 1 min, indicating that the “swelling-collapsing” transformation of PMAA molecules was rapid. This observation was consistent with known conformational change of PMAA molecules in response to pH change. The PMAA molecules presented an extensively swelled conformation in basic aqueous solution because of the ionization of the carboxyl groups (COOH) into carboxylate ions (COO^-^), which resulted in a high density of negative charges, strong electrostatic repulsion, and a high degree of hydration. In acidic aqueous solution, the PMAA molecules were protonated and collapsed due to the decrease of electrostatic repulsion 56, 57. In addition to the fluorescent method, the “swelling-collapsing” transformations of PMAA molecules were also confirmed by measuring the water contact angle (Figure 2E and F). For Ti-NTs, Ti-NTs-D and Ti-NTs-PCtrl, there were negligible changes in contact angles with changes in pH (Figure 2E). However, the contact angle of Ti-NTs-P increased from 12.8° at pH 7.4 to 27.6° at pH 5.0 (Figure 2E), due to the transformation of COO^-^ toward COOH and the hiding of the hydrophilic groups (i.e., COOH) in the collapsed PMAA molecules when pH was decreased 40. Meanwhile, this transformation could be fully repeated for at least 5 cycles, demonstrating the recyclable “collapsing-swelling” ability of PMAA (Figure 2F).

### Release kinetics of AMPs from the substrates

**Figure 3:**
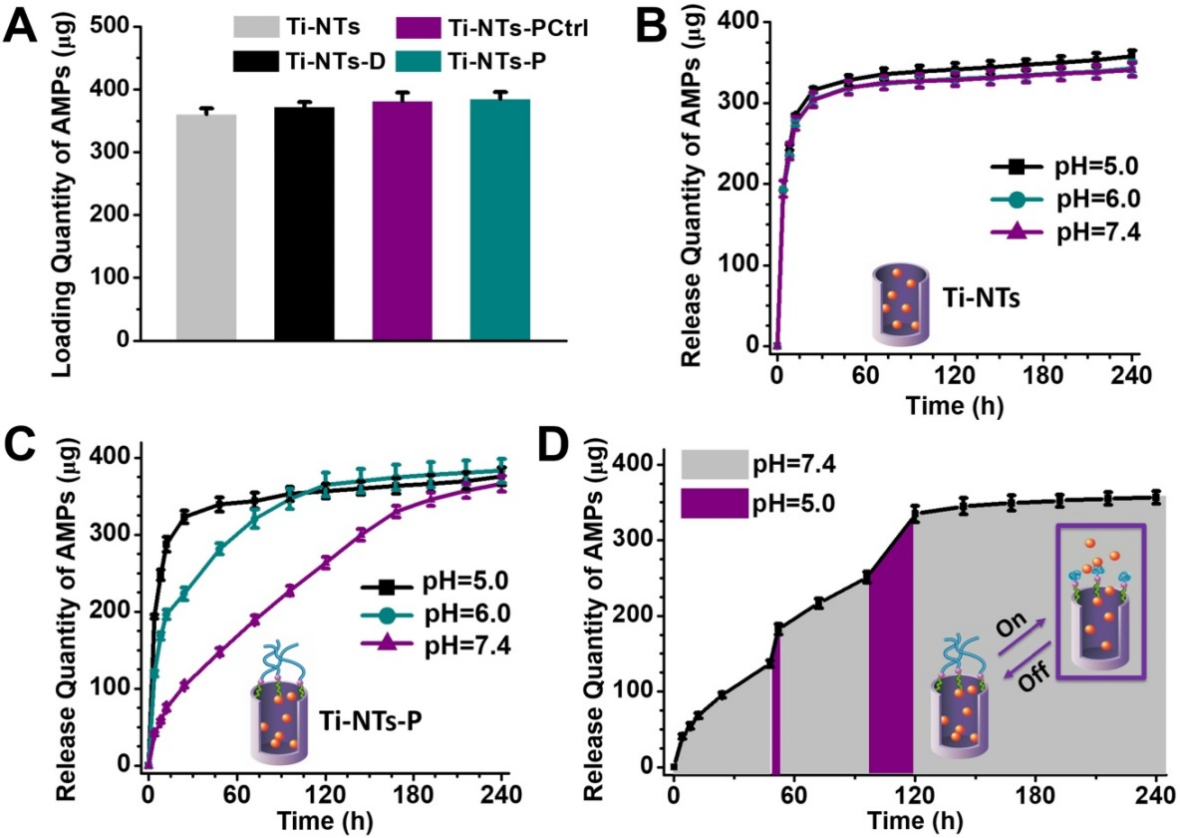
** The loading and release of HHC36 peptides by the indicated substrates.** (A) The quantity of HHC36 peptides loaded on the indicated substrates. (B) The release of HHC36 peptides from Ti-NTs-A at different pH values between 7.4 to 5.0. (C) The release of HHC36 peptides from Ti-NTs-P-A at different pH values between 7.4 to 5.0. (D) The release of HHC36 peptides from Ti-NTs-P-A with a change in pH values at random time points. In the present study, 48 h and 96 h were chosen as the random time points. All error bars denote the standard deviations over triplicate measurements with separately implants.

To load HHC36 peptides on the implants, particularly on Ti-NTs-P, we decreased the pH to 5.0 to “open” the PMAA gates (Scheme 1B). According to the maximum absorption peak and the fitting curve of HHC36 peptides (Figure S4, SI), the quantity of HHC36 peptides loaded on Ti-NTs-P was estimated to be 384.7 μg/cm^2^ (Figure 3A). This quantity was similar to those of Ti-NTs (360.4 μg/cm^2^), Ti-NTs-D (372.5 μg/cm^2^) and Ti-NTs-PCtrl (381.5 μg/cm^2^) (Figure 3A), demonstrating that PMAA gates did not impact the loading of peptides. Herein, to distinguish the surfaces with/without HHC36 peptides, we denoted the surfaces, Ti-NTs, Ti-NTs-D, Ti-NTs-PCtrl and Ti-NTs-P, as Ti-NTs-A, Ti-NTs-D-A, Ti-NTs-PCtrl-A and Ti-NTs-P-A after AMPs loading (Scheme 1).

We characterized the release of AMPs from these surfaces. In the present study, we set the time in which 50% of the peptides were released as “50%-release time”, and the time in which 90% of the peptides were released as “90%-release time”. In a physiological environment (pH = 7.4), burst releases of AMPs from the control groups occurred, and the 50%-release times of Ti-NTs-A, Ti-NTs-D-A and Ti-NTs-PCtrl-A were less than or approximately 4 h. In total, 68.6%, 64.0% and 65.1% of AMPs were released from Ti-NTs-A, Ti-NTs-D-A and Ti-NTs-PCtrl-A in the first 8 h, respectively. The 90%-release times of these three surfaces were approximately 24 h (Figure 3B, Figure S5 and Figure S6, SI). The results obviously indicated the burst release of AMPs in these control groups. Moreover, the release rates of these control groups had no relationship to the pH of solution.

Different from those control surfaces, Ti-NTs-P-A showed sustainable release of AMPs for a long time (Figure 3C). In the physiological environment (pH = 7.4), a little burst release occurred in the initial stage, and 14.7% of the AMPs was released in the first 8 h. It is known that the burst release plays an important role in this stage due to the weakened immune system 9, 12. Namely, in approximately 6-8 h after surgery, adequate release of AMPs is desired to kill the bacteria because of the weakened immunological system. After this stage, the release rate became slow, and only 28.4% of the AMPs were released after 24 h. Compared to those of Ti-NTs, the 50%-release time and 90%-release time of Ti-NTs-P-A could be nearly 20-fold and 7-fold greater, i.e., approximately 72 h and 168 h, respectively. The whole release time of Ti-NTs-P-A lasted up to 7 to 10 days, which was consistent with the acute infection period in the clinic.

In contrast to other studies, the release of AMPs from Ti-NTs-P-A could be stimulated by an infection microenvironment (acidic pH) via the opening of PMAA molecular “gates” (Figure 3C). When the pH decreased from 7.4 to 6.0 or 5.0 to simulate a severe infection microenvironment, 42.3% (2.9-fold greater than that in a physiological environment) or 62.3% (4.3-fold greater than that in a physiological environment) of the AMPs were released in 8 h. This demonstrated that the surface could release sufficient AMPs in response to an infection microenvironment. Furthermore, it was of great significance that such smart response could occur at any stage of the release process (Figure 3D). If the pH changed from 7.4 to 5.0 at random time points (e.g., at 48 h and 96 h), the burst release could be observed (Figure 3D).

In fact, we also found that when AMPs were replaced by a protein such as BSA or an antibiotic such as doxycycline hyclate (Doxy), the on-demand release was also effective (Figure S7 - S14, Table S3, SI), demonstrating that our on-demand delivery and release strategy could be extended to other target molecules. Additionally, we demonstrated that the loading amount (mole) of the drugs in the nanotubes would increase with the reduction of the molecular weight of the drugs, as the larger molecule could occupy more space in the nanotubes. For example, on Ti-NTs, the loading amount of Doxy (488.2 ± 8.9 nmol/cm^2^) was higher than that of HHC36 (242.2 ± 12.3 nmol/cm^2^) and BSA (128.0 ± 3.0 nmol/cm^2^).

### Bactericidal activities of the “gates” engineered Ti-NTs substrates

**Figure 4:**
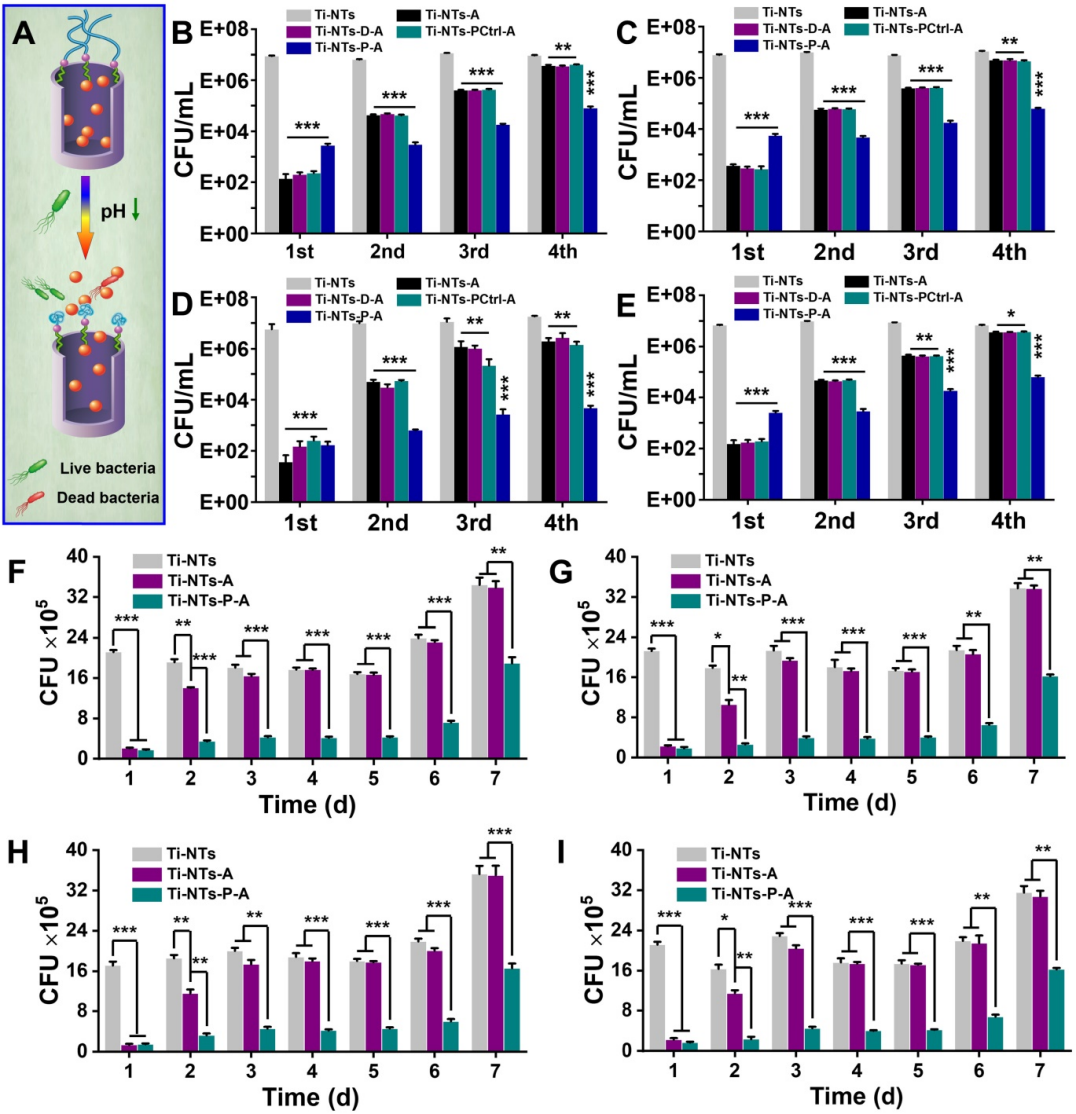
** The *in vitro* bactericidal activities of the substrates.** (A) Schematic diagram of the bactericidal activities of Ti-NTs-P-A at different pH values, which were controlled via the “swelling-collapsing” of PMAA molecules. (B-E) Bactericidal activity of the indicated substrates against (B) *S. aureus*, (C) *E. coli*, (D) *P. aeruginosa* and (E) *MRSA* in 1 h for four cycles. (F-I) The sustained bactericidal activity of the indicated substrates against (F) *S. aureus*, (G) *E. coli*, (H) *P. aeruginosa* and (I) *MRSA* in 7 days. The substrates were immersed in PBS for the specified times before tested. * denotes *p* < 0.05, ** denotes *p* < 0.01, *** denotes *p* < 0.001. All error bars denote the standard deviations over triplicate measurements with separately implants.

The MIC_90_ and MBC values of the AMPs (in solution) against *S. aureus*, *E. coli*, *P. aeruginosa* and *MRSA* were 12.5 μM, 10 μM, 7.5 μM, 12.5 μM (MIC_90_) (Figure S15, SI), and 25 μM, 25 μM, 15 μM, 25 μM (MBC), respectively, demonstrating its excellent bactericidal activity. The bactericidal activities of AMPs-loaded titanium surfaces were then examined by counting the colony forming units (CFUs) of surviving bacteria after culturing (Figure 4A - E). In the first cycle, all the control groups with AMPs exhibited credible bactericidal activity with more than 99.99% bacterial decrease (e.g., 6.3 × 10^4^-fold, 2.1 × 10^4^-fold, 1.5 × 10^5^-fold and 4.5 × 10^4^-fold decrease of *S. aureus*, *E. coli*, *P. aeruginosa* and *MRSA* on Ti-NTs-A relative to Ti-NTs without AMPs). Ti-NTs-D-A and Ti-NTs-PCtrl-A had similar bactericidal activities as Ti-NTs-A. Although the bactericidal activity of Ti-NTs-P-A was slightly lower than the three control groups due to its low release rate of peptides at the initial stage, it still inhibited 99.9% of bacterial growth (3.1 × 10^3^-fold, 1.4 × 10^3^-fold, 3.4 × 10^4^-fold and 2.7 × 10^3^-fold decrease of *S. aureus*, *E. coli*, *P. aeruginosa* and *MRSA* on Ti-NTs-P-A relative to Ti-NTs-P). This finding also revealed that an adequate amount of AMPs could be released from Ti-NTs-P-A at the initial stage, when infection occurred.

However, compared to other groups, Ti-NTs-P-A showed more stable bactericidal activity in the subsequent rounds. For example, in the fourth round, compared to Ti-NTs, Ti-NTs-A killed only 60.0%, 54.5%, 91.2% and 47.4% of *S. aureus*, *E. coli*, *P. aeruginosa* and *MRSA*, respectively. In contrast, Ti-NTs-P-A still killed more than 99% of bacteria in this cycle (120-fold, 170-fold, 3800-fold and 110-fold decreases of *S. aureus*, *E. coli*, *P. aeruginosa* and *MRSA* in Ti-NTs-P-A relative to Ti-NTs-P, respectively). The antimicrobial results were in accordance with the release rate of HHC36 peptides in the indicated substrates, demonstrating an improvement in continuous bactericidal activity by grafting PMAA molecules.

We further characterized the sustainable bactericidal activity of the indicated substrates (Figure 4F - I) over 7 days. After being exposed to PBS for 1 day, Ti-NTs-A and Ti-NTs-P-A showed excellent bactericidal activity in 4.2 h and inhibited 90.1% and 91.8% of *S. aureus*, 89.3% and 91.4% of *E. coli*, 92.4% and 91.7% of *P. aeruginosa*, and 89.5% and 92.1% of *MRSA*, respectively. However, the bactericidal activity of Ti-NTs-A disappeared completely from the 3^rd^ day, while that of Ti-NTs-P-A could be maintained for 7 days. After being released under physiological conditions for 6 and 7 days and then allowed to be exposed to bacteria, Ti-NTs-P-A could still inhibit 70.1% and 45.1% of *S. aureus*, 69.6% and 51.8% of *E. coli*, 72.8% and 52.9% of *P. aeruginosa*, and 69.0% and 48.5% of *MRSA* in 6.8 h and 8 h, respectively (Figure 4F - I). These results were consistent with the release rate of the AMPs from the indicated substrates, demonstrating that Ti-NTs-P-A could show sustainable and on-demand bactericidal activity during the acute infection period. Since our strategy was different from that of extending the validity time by restricting the release of AMPs, Ti-NTs-P-A showed better bactericidal activity by adequately releasing AMPs via pH-stimulated properties. We also characterized the bactericidal activities of AMPs and the samples after steam sterilization for further clinical application, and found that the bactericidal activities of AMPs and the samples against *S. aureus* were not compromised by steam sterilization (Figure 4B, Figure S16 and S17, SI).

### Cytotoxicity and osteogenic activities of the “gates” engineered Ti-NTs *in vitro*

**Figure 5:**
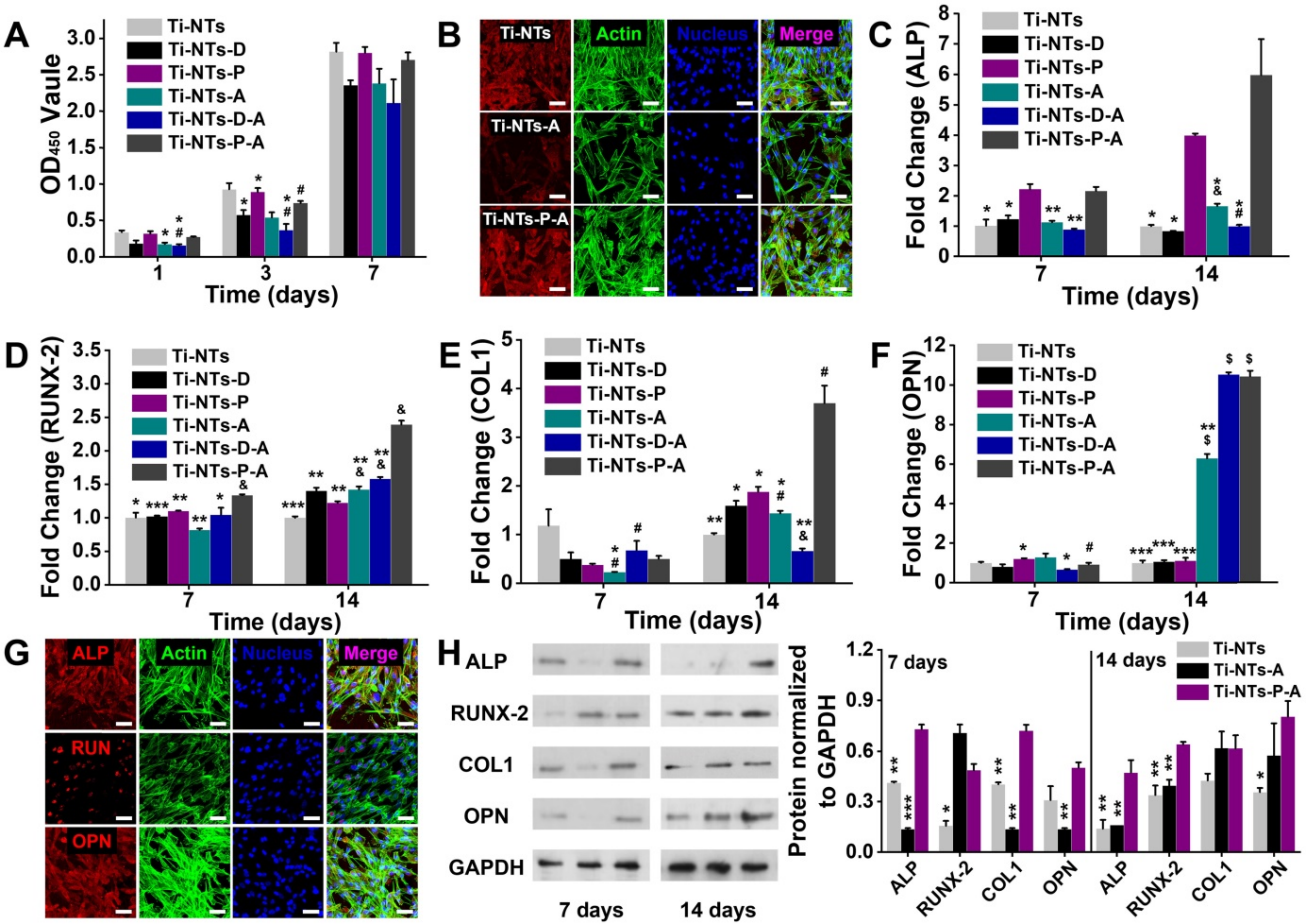
***In vitro* biocompatibilities and osteogenic activities of the substrates.** (A) CCK-8 assay of* hBMSCs* on the indicated surfaces after 1, 3 and 7 days of culture. * denotes *p* < 0.05 and ** denotes *p* < 0.01 compared to Ti-NTs, # denotes *p* < 0.05 compared to the corresponding control group without peptides. Error bars denote the standard deviations over quadruplicate measurements with separately implants. (B) Confocal fluorescence microscopy images of *hBMSCs* stained with vinculin, F-actin and DAPI after being cultured for 24 h. Scale bar, 50 μm. (C-F) qRT-PCR assay of osteogenic gene expression of (C) ALP, (D) RUNX-2, (E) COL1 and (F) OPN of *hBMSCs* after 7 and 14 days of culture. * denotes *p* < 0.05, ** denotes *p* < 0.01 and *** denotes *p* < 0.001 compared to Ti-NTs-P-A; # denotes *p* < 0.05, & denotes *p* < 0.01 and $ denotes *p* < 0.001 compared to the corresponding control group without peptides. All error bars denote the standard deviations over quadruplicate measurements with separately implants. (G) Immunofluorescence staining of *hBMSCs* cultured on Ti-NTs-P-A for 7 days (ALP and RUNX-2) and 14 days (OPN). The images were obtained by confocal fluorescence microscopy. Scale bar, 50 μm. (H) Western blotting of *hBMSCs* cultured on the substrates for 7 and 14 days. At each time point, left lane was Ti-NTs, middle lane was Ti-NTs-A and right lane was Ti-NTs-P-A. * denotes *p* < 0.05, ** denotes *p* < 0.01 and *** denotes *p* < 0.001 compared to Ti-NTs-P-A. Error bars denote the standard deviations over triplicate measurements with separately Western blotting results.

The cytotoxicity of the indicated substrates was quantitatively evaluated by CCK-8 assay (Figure 5A). Compared to Ti-NTs, Ti-NTs-D (with integrated dopamine) exhibited cytotoxicity, and Ti-NTs-P (i.e., Ti-NTs-D with PMAA immobilized) shielded dopamine by PMAA to restore the biocompatibility in 7 days. After the loading of AMPs, Ti-NTs-A and Ti-NTs-D-A exhibited significantly higher cytotoxicity than Ti-NTs and inhibited 47.1% and 52.6% of *hBMSCs*, respectively, on the 1^st^ day. These results were consistent with previous reports 35, 36, and the cytotoxicity was induced by the burst release of AMPs. After the burst release, the proliferation rates of the cells in these two groups became similar to those of the control groups on day 7, demonstrating the exhaustion of the AMPs supply. However, the biocompatibility could be strongly improved by controlling the release of AMPs. The biocompatibility of Ti-NTs-P-A increased 1.5-fold compared to that of Ti-NTs-A and was similar to that of Ti-NTs (0.8-fold) and Ti-NTs-P (0.9-fold) on the 1^st^ day. Even though the sustained release lasted for more than 7 days in physiological conditions (Figure 3C), the biocompatibility of Ti-NTs-P-A was similar to those of Ti-NTs and Ti-NTs-P during this period.

Fluorescence images also provided direct evidence of the adhesion and spreading of the cells on different surfaces (Figure 5B and Figure S18, SI). The cells adhered to Ti-NTs, Ti-NTs-P and Ti-NTs-P-A, and displayed well spreading according to the actin staining (green) and the vinculin staining (red). In contrast, on Ti-NTs-A and Ti-NTs-D-A, the cell adhesion was reduced according to the actin staining. The analogous vinculin staining and DAPI staining showed that the red fluorescence was concentratedly distributed around the cell nucleus, demonstrating that the cell spreading was restricted. These fluorescent results were consistent with the CCK-8 assays (Figure 5A) and suggested that Ti-NTs-P-A had higher biocompatibility for cell adhesion and spreading compared to other bactericidal groups.

Moreover, we characterized the indicators, including ALP, RUNX-2, COL1 and OPN of osteogenic differentiation of *hBMSCs* on different surfaces by qRT-PCR (Figure 5C - F). Osteogenic differentiation can be divided into three major periods 58, 59: proliferation with the expression of ALP 58, matrix deposition in which the RUNX-2 stimulated the activity of the collagen 1 promoter fragment to increase the deposition 60, 61, and mineralization with the expression of OPN in later stage 58, 62, 63. On the 7^th^ day and compared to the control group (Ti-NTs-P), the expression of the genes would not be limited on Ti-NTs-P-A. The expressions of ALP and RUNX-2 on Ti-NTs-P-A were higher than other groups, indicating the accelerating cell proliferation and enhanced promoter fragment of matrix deposition. On the 14^th^ day, all the osteogenic genes on Ti-NTs-P-A had higher expression compared to other groups (except that the expression of OPN on Ti-NTs-P-A was similar to that on Ti-NTs-D-A). HHC36 peptides had been proved to maintain 7 or even enhance 64 the osteointegration with appropriate concentrations. Herein, we further showed that by controlling the release, this AMPs can enhance the expression of related osteogenic genes. Additionally, the burst release of the AMPs would impact the expression of osteogenic genes on the 7^th^ day. Compared to their own control groups (Ti-NTs and Ti-NTs-D), the expressions of RUNX-2/COL1 on Ti-NTs-A and the expression of ALP on Ti-NTs-D-A were suppressed. The expressions of genes on Ti-NTs-A and Ti-NTs-D-A gradually recovered until the 14^th^ day. However, the promotion effect of AMPs to the expression of osteogenic genes was limited as the exhausting of the peptides.

Further immunofluorescence staining and Western blotting assays of ALP, RUNX-2 and OPN showed the same trends as qRT-PCR, suggesting the enhanced expression of the related proteins on Ti-NTs-P-A and the suppressed expression of the related proteins on Ti-NTs-A and Ti-NTs-D-A (Figure 5G and H, Figure S19 to Figure S21, SI). The above results demonstrated that the controlled release of AMPs on Ti-NTs-P-A could enhance the osteogenic differentiation of *hBMSCs*.

### Bactericidal activities and biocompatibilities of the “gates” engineered Ti-NTs* in vivo*

**Figure 6:**
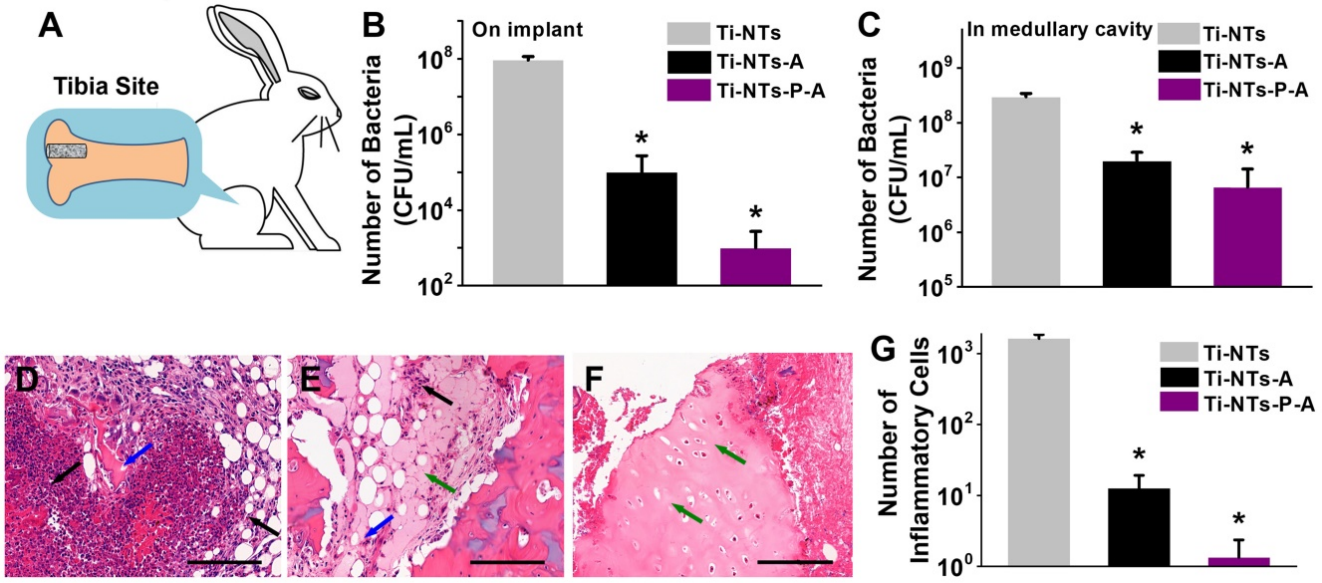
***In vivo* bactericidal activities and biocompatibilities of the substrates.** (A) Implant site in the tibia bone of New Zealand rabbit. Bactericidal activity on the implant surface (B) and in the medullary cavity (C). All data are expressed as means ± SD over triplicate experiments by blood agar plates. (D-F) The photomicrographs of longitudinal sections of the proximal tibia of rabbits treated with (D) Ti-NTs, (E) Ti-NTs-A and (F) Ti-NTs-P-A and stained by hematoxylin and eosin (H&E). The black, blue and green arrows indicated inflammatory cells, connective tissue and osteoblasts, respectively. Scale bar, 200 μm. (G) The number of inflammatory cells around the indicated groups. * denotes *p* <0.001. Error bars denote the standard deviations over sextuplicate measurements with aliquots of the same samples.

Furthermore, we evaluated the bactericidal activity and biocompatibility of the indicated implants *in vivo*. We chose *S. aureus* as the basis for building a bacterial infection/bone defect model, as it accounts for two-thirds of infective bacteria in the clinic 65-67 (Figure 6A). After 7 days, Ti-NTs-A and Ti-NTs-P-A killed 99.89% and 99.99% of *S. aureus* on their surface, respectively, compared to Ti-NTs (Figure 6B). Additionally, in contrast to our previous studies in which covalently immobilized AMPs on Ti surfaces showed poor bactericidal activity against untouched bacteria 42, Ti-NTs-A and Ti-NTs-P-A inhibited 93.30% and 97.76% of *S. aureus* in the medullary cavity, respectively (Figure 6C). We also found that Ti-NTs-P-A had better bactericidal activity than Ti-NTs-A, demonstrating that the improvement in the release of AMPs efficiently enhanced the inhibition of bacterial infection.

We employed the H&E (hematoxylin and eosin) staining to investigate the tibia bone's pathology (Figure 6D - F). Around Ti-NTs, there were large amounts of inflammatory cells, suggesting the serious inflammatory reaction (Figure 6D). And the inflammatory reaction around Ti-NTs-A decreased evidently due to its antimicrobial activity (Figure 6E). In particularly, although only 97.76% of *S. aureus* (not 99.99%) in the medullary cavity were killed by Ti-NTs-P-A, it seemed that the residual bacteria were insufficient to cause serious infection, while negligible histological infection and bone destruction could be observed around Ti-NTs-P-A (Figure 6F). The quantified results showed that the amount of inflammatory cells was 99.2% lower in Ti-NTs-A than that in Ti-NTs, while that in Ti-NTs-P-A was the lowest (99.9% lower than that in Ti-NTs) (Figure 6G). Further analysis showed that compared to Ti-NTs-A, Ti-NTs-P-A had negligible connective tissues (Figure 6E and F), and the number of inflammatory cells decreased by 9.5-fold (Figure 6G), demonstrating that Ti-NTs-P-A displayed better biocompatibility due to the improved burst release of AMPs in bacterial infection environment. Consequently, we believe this system holds great potential in developing antimicrobial titanium implants for orthopaedic patients.

## Conclusions

In summary, we demonstrated the design of a smart system on titanium implants for sustainable and on-demand delivery of AMPs. PMAA molecules, which served as the switchable gates, were responsible for the pH-stimulated delivery of HHC36 peptides. Under physiological conditions, the PMAA molecules could swell to close the opening of the nanotubes and thus reduce the release of AMPs, and the release time could be extended from dozens of hours to 10 days. More importantly, when pH values decreased to simulate infection conditions, the PMAA molecules collapsed to open the nanotubes and thus release an adequate amount of peptides to ultimately kill bacteria. The system had sustained and on-demand bactericidal activity, improved biocompatibility and osteogenic activity *in vitro*. The *in vivo* assay also demonstrated that the system inhibited bacterial infection and exhibited biocompatibility during the acute infection period after implantation. Furthermore, this strategy exhibited a broad-spectrum application for the delivery of other biomolecules (e.g., BSA in the present study) from titanium implants. The findings will be of great interest for sustained and on-demand drug delivery on titanium implants for orthopaedic patients.

## Supplementary Material

Supplementary experimental section, figures, and tables.

## Figures and Tables

**Table 1:** The abbreviations of the samples

Sample abbreviation	Treatment method
Ti-NTs	Ti substrate treated with anodization
Ti-NTs-D	Ti-NTs treated with dopamine solution
Ti-NTs-PCtrl	Ti-NTs treated with PMAA solution
Ti-NTs-P	Ti-NTs-D treated with PMAA solution
Ti-NTs-A	Ti-NTs loaded with HHC36 peptides
Ti-NTs-D-A	Ti-NTs-D loaded with HHC36 peptides
Ti-NTs-PCtrl-A	Ti-NTs-PCtrl loaded with HHC36 peptides
Ti-NTs-P-A	Ti-NTs-P loaded with HHC36 peptides
