# Supplementary material for: On-demand storage and release of antimicrobial peptides using Pandora's box-like nanotubes gated with a bacterial infection-responsive polymer
Source: Theranostics. 2020 Jan 1;10(1):109–22. doi: 10.7150/thno.38388 (PMC6929614; doi:10.7150/thno.38388)
Supplement: Supplementary file 1 — Supplementary experimental section, figures, and tables. [file thnov10p0109s1.pdf]

Supporting Information

On-demand storage and release of antimicrobial peptides using Pandora's box-like nanotubes gated with a bacterial infection-responsive polymer

Junjian Chen[1,4,†], Xuetao Shi[2,5,†], Ye Zhu[3], Yunhua Chen[1,5], Meng Gao[1,6], Huichang Gao[1,4], Lei Liu[2,6], Lin Wang[2,6,*], Chuanbin Mao[3,*], Yingjun Wang[1,4,6,*]

1. National Engineering Research Center for Tissue Restoration and Reconstruction, South China University of Technology, Guangzhou 510006, China. Email: imwangyj@scut.edu.cn

2. Key Laboratory of Biomedical Materials and Engineering of the Ministry of Education, South China University of Technology, Guangzhou 510641, China.

3. Department of Chemistry and Biochemistry, University of Oklahoma, Stephenson Life Sciences Research Center Norman, OK, 73019, USA. E-mail: cbmao@ou.edu

4. School of Biomedical Science and Engineering, South China University of Technology, Guangzhou 510006, China.

5. Key Laboratory of Biomedical Engineering of Guangdong Province, South China University of Technology, Guangzhou 510006, China.

6. Guangzhou Regenerative Medicine and Health Guangdong Laboratory, Guangzhou 510006, China. Email: wanglin3@scut.edu.cn

† These authors contributed equally to this work.

KEYWORDS: Titania nanotubes, pH-Responsive molecular gate, On-demand delivery, Bactericidal activity, Peptides

Supplemental experimental section

Characterization of the PMAA transformation with AIEgens: "Swelling-collapsing" transformation of PMAA "gate" was evaluated by grafting aggregation-induced emission luminogens (AIEgens, TPE-NH_2, acquired from Prof. Ben Zhong Tang' groups as in reference [1]) on Ti-NTs-P via an amide coupling reaction. The structure of TPE-NH_2 was shown below. Briefly, Ti-NTs-P was immersed in a mixed solution containing 50 mM WSC, 20 mM NHS and 5 mM TPE-NH_2 for 24 h. The change of fluorescence intensity was characterized in the PBS with different pH values. We dropped PBS (pH = 7.4) on the surface of samples, waited for 1 min, and then tested the MFI by Leica DigitalMicroscope (DM6M-Z, Germany). After that, we removed the residual solution, washed the surface five times with distilled water, dropped PBS (pH = 5.0, adjusting by hydrochloric acid) on the surface of samples, waited for 1 min, and then tested the MFI. The above processes were repeated 5 times.

The structure of TPE-NH_2:

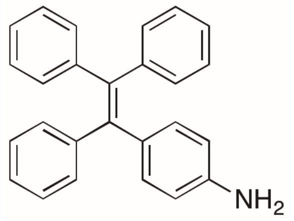

Colorimetric staining with Acid Orange II: The amine concentration of Ti-NTs-D was determined by Acid Orange II (AO) according to other references [2-4]. Briefly, Ti-NTs and Ti-NTs-D were immersed into a 500 μM AO solution with pH 3, set by HCl. After shaken for 12 h at room temperature, the substrates were washed twice with acidic water. Then, the substrates were shaken for 15 min in water with pH 12 (set by NaOH) to dissolve the adsorbed AO. The AO concentration (which was similar to the amine concentration of the substrates) of the solution was colorimetrically determined with an optical spectrometer at 485 nm.

The exact grafting amount of PMAA: The quantity of the grafted PMAA was calculated according to the OD_{211} values of the original solution and the cleaning

solution as follows (Figure S3):

$$y = 0.0055x + 0.0602, \qquad R^2 = 0.9998$$

$$m_l = x_o V_o - x_{cs} V_{cs}$$

where y is the OD_{211} value, and x is the concentration of PMAA in μM; m_l is the quantity of PMAA; X_o is the concentration of PMAA in original solution; V_o is the volume of original PMAA solution; x_{cs} is the concentration of the PMAA in the post-reacted and cleaning solution; V_{cs} is the volume of the post-reacted and cleaning solution.

The post-reacted and cleaning solution was dialyzed for 24 h to remove EDC and NHS before tested.

The quantity of the loaded HHC36 peptides: The quantity of the loaded HHC36 peptides was calculated according to the OD_{280} values of the original solution and the cleaning solution as follows:

$$y = 2.2936x - 0.0121, \qquad R^2 = 0.9998$$

$$m_l = x_o V_o - x_{cs} V_{cs}$$

where y is the OD_{280} value, and x is the concentration of the HHC36 peptides in mM; m_l is the quantity of the loaded HHC36 peptides; x_o is the concentration of the HHC36 peptides in original solution; V_o is the volume of original HHC36 peptides solution; x_{cs} is the concentration of the HHC36 peptides in the post-reacted and cleaning solution; V_{cs} is the volume of the post-reacted and cleaning solution.

Loading and releasing of BSA: Samples (Ti-NTs, Ti-NTs-D, Ti-NTs-PCtrl and Ti-NTs-P) were placed in 24-well plates. Then, 50 μL of a BSA solution (1 mM in a mixture of ethanol and water, $V_{ethanol}$: V_{water} = 1:1, pH = 5.0) was added on the substrates. The substrates were dried under a vacuum desiccator and repeated 10 times. The substrates were cleaned with PBS, and the quantity of BSA loaded was calculated according to the OD_{278} values of the original solution and the cleaning solution as follows:

$$y = 0.009x - 0.0055, \qquad R^2 = 0.9999$$

$$m_l = x_o V_o - x_{cs} V_{cs}$$

where y is the OD_{278} value, and x is the concentration of the BSA in μM; m_l is the

quantity of the loaded BSA; x_o is the concentration of the BSA in original solution; V_o is the volume of original BSA solution; x_{cs} is the concentration of the BSA in the post-reacted and cleaning solution; V_{cs} is the volume of the post-reacted and cleaning solution.

The samples with BSA were placed in 24-well plates, and 250 μL of PBS was injected into each well. After culturing (37 °C), the solution was collected and refreshed PBS was added at the indicated time points. Then, the rate of the BSA release from different substrates was calculated by measuring the UV absorption of the collected solution at OD_{278}. We also characterized the controlled release of BSA in different microenvironments by adjusting the pH values of the PBS with 0.1 M HCl solution.

Based on the maximum absorption peak and the fitting curve of BSA (Figure S7), we found that the quantity of BSA loaded on Ti-NTs, Ti-NTs-D, Ti-NTs-PCtrl and Ti-NTs-P was 8.5, 9.0, 8.9 and 8.7 mg/cm^2, respectively (Figure S8). Meanwhile, BSA release rates resembled the HHC36 peptides release rates in that the slow, sustainable and on-demand release of BSA could be observed from Ti-NTs-P (Figure S9 and Figure S10).

Loading and releasing of doxycycline hyclate (Doxy): Samples (Ti-NTs, Ti-NTs-D, Ti-NTs-PCtrl and Ti-NTs-P) were placed in 24-well plates. Then, 50 μL of a Doxy solution (1 mM in methanol (512.9 μg/mL), and the pH was adjusted to 5.0 by HCl solution for collapsing the PMAA molecules) was added onto the substrates. The substrates were dried under a vacuum desiccator and repeated 20 times. After that, the substrates were cleaned with PBS, and abbrivated as Ti-NTs-Doxy, Ti-NTs-D-Doxy, Ti-NTs-PCtrl-Doxy and Ti-NTs-P-Doxy, respectively (Table S3). The quantity of Doxy loaded was calculated according to the OD_{350} values of the original solution and the cleaning solution as follows:

$$y = 0.0029x - 0.0043, \qquad R^2 = 0.9999$$

$$m_l = x_o V_o - x_{cs} V_{cs}$$

where y is the OD_{350} value, and x is the concentration of the Doxy in μM; m_l is the quantity of the loaded Doxy; x_o is the concentration of the Doxy in original solution; V_o is the volume of original Doxy solution; x_{cs} is the concentration of the Doxy in the

post-reacted and cleaning solution; V_{cs} is the volume of the post-reacted and cleaning solution.

Based on the maximum absorption peak and the fitting curve of Doxy, we found that the quantity of Doxy loaded on Ti-NTs, Ti-NTs-D, Ti-NTs-PCtrl and Ti-NTs-P was 250.4, 248.5, 248.1 and 247.6 μg/cm^2, respectively (Figure S11). The samples were placed in 24-well plates, and 250 μL of PBS was added into each well at 37 °C. The incubation solution was collected at the indicated time points. Then, the rate of Doxy was calculated by measuring the UV absorption of the collected solution at OD_{350}. We also characterized the release of Doxy in different pH solution (Figure S12 and Figure S13).

***In vitro* bactericidal assay of the surface with Doxy:** 250 μL of bacteria suspension, diluted in LB medium (1 × 10^7 CFU/mL), was added to fully cover the surface. After 1 h in culture, the bacterial suspension was immediately transferred from the 24-well plate to Eppendorf tubes, and each suspension was taken to streak onto agar plates. The amount of bacteria was determined after 15 h. The substrates were air-dried, and the above procedures were repeated 3 more times (Figure S14).

References:

1. Song ZG, Mao D, Sung SHP, Kwok RTK, Lam JWY, Kong DL, et al. Activatable fluorescent nanoprobe with aggregation-induced emission characteristics for selective in vivo imaging of elevated peroxynitrite generation. Adv Mater. 2016; 28: 7249-56.
2. Uchida E, Uyama Y, Ikada Y. Sorption of low molecular-weight anions into thin polycation layers grafted onto a film. Langmuir. 1993; 9: 1121-4.
3. Hamerli P, Weigel T, Groth T, Paul D. Surface properties of and cell adhesion onto allylamine-plasma-coated polyethylenterephtalat membranes. Biomaterials. 2003; 24: 3989-99.
4. Qiu H, Qi P, Liu J, Yang Y, Tan X, Xiao Y, et al. Biomimetic engineering endothelium-like coating on cardiovascular stent through heparin and nitric oxide-

generating compound synergistic modification strategy. Biomaterials. 2019; 207: 10-22.

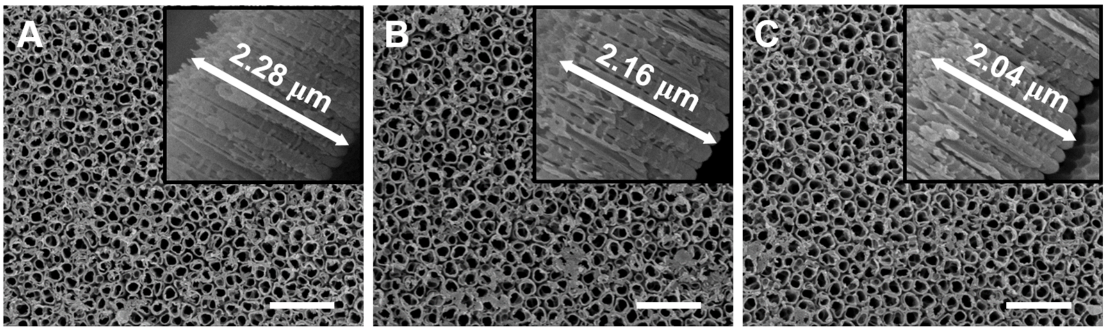

Figure S1. SEM images of the Ti-NTs-D (A), Ti-NTs-P (B) and Ti-NTs-A (C). Inset: cross-section view. Scale bar, 500 nm. See Section *Surface characterization* for details of the assay.

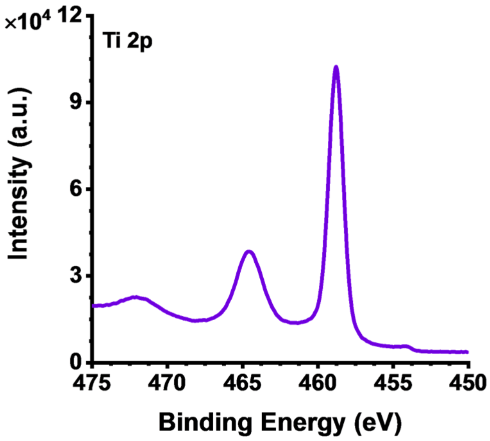

Figure S2. High-resolution XPS Ti 2p spectra of Ti-NTs-PCtrl. See Section *Surface characterization* for details of the assay.

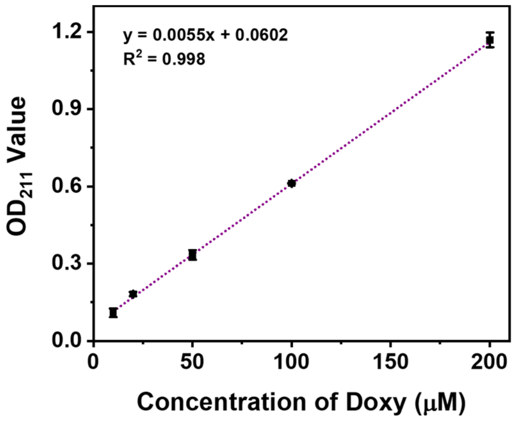

Figure S3. The fitting curve of OD_{211} versus concentration of PMAA. See Section *The exact grafting amount of PMAA* for details of the assay. Error bars denote the standard deviations over triplicate measurements with separate implants.

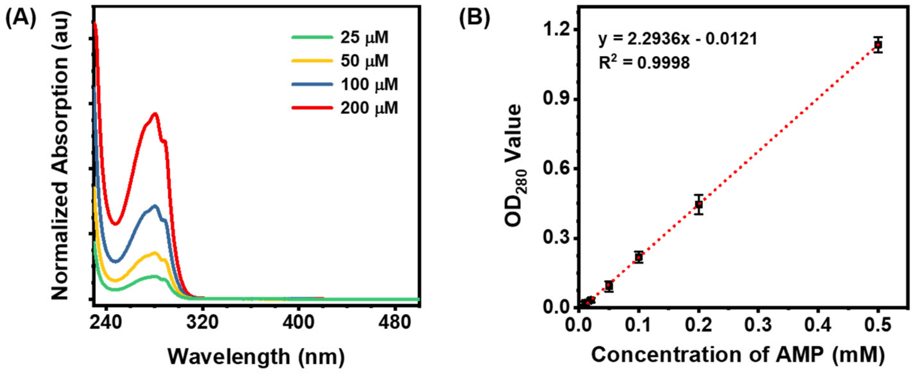

Figure S4. (A) Normalized UV-vis absorption of different concentrations of HHC36 peptides in aqueous solution. According to the absorption peak, we chose the wavelength of 280 nm to measure the OD values. (B) The fitting curve of OD_{280} versus concentration of HHC36 peptides. See Section *Surface characterization* for details of the assay. Error bars denote the standard deviations over triplicate measurements with separate implants.

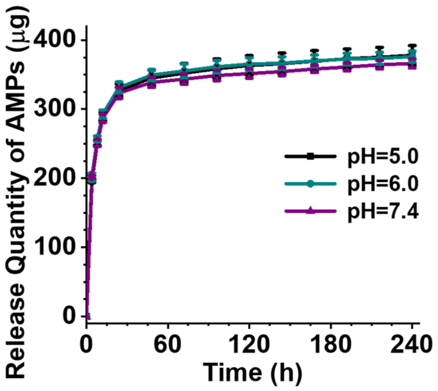

Figure S5. The release of HHC36 peptides from Ti-NTs-D at different pH values. See Section *Loading and releasing of HHC36 peptides* in main text for details of the assay. Error bars denote the standard deviations over triplicate measurements with separate implants.

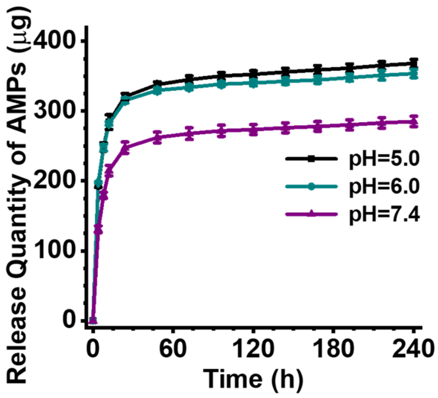

Figure S6. The release of HHC36 peptides from Ti-NTs-PCtrl at different pH values. See Section *Loading and releasing of HHC36 peptides* in main text for details of the

assay. Error bars denote the standard deviations over triplicate measurements with separate implants.

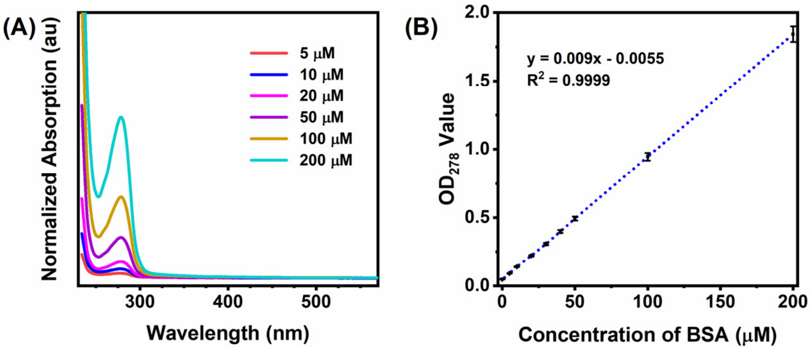

Figure S7. (A) Normalized UV-vis absorption of different concentrations of BSA in aqueous solution. According to the absorption peak, we chose the wavelength of 278 nm to measure the OD values. (B) The fitting curve of OD_{278} versus the concentrations of BSA. See Section *Surface characterization* for details of the assay. Error bars denote the standard deviations over triplicate measurements with separate implants.

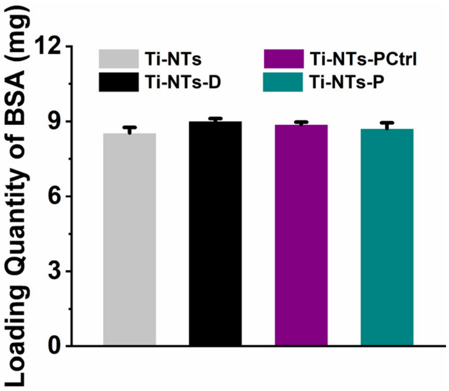

Figure S8. The quantity of BSA loaded in the indicated substrates. See Section *Loading and releasing of BSA* for details of the assay. Error bars denote the standard deviations over triplicate measurements with separate implants.

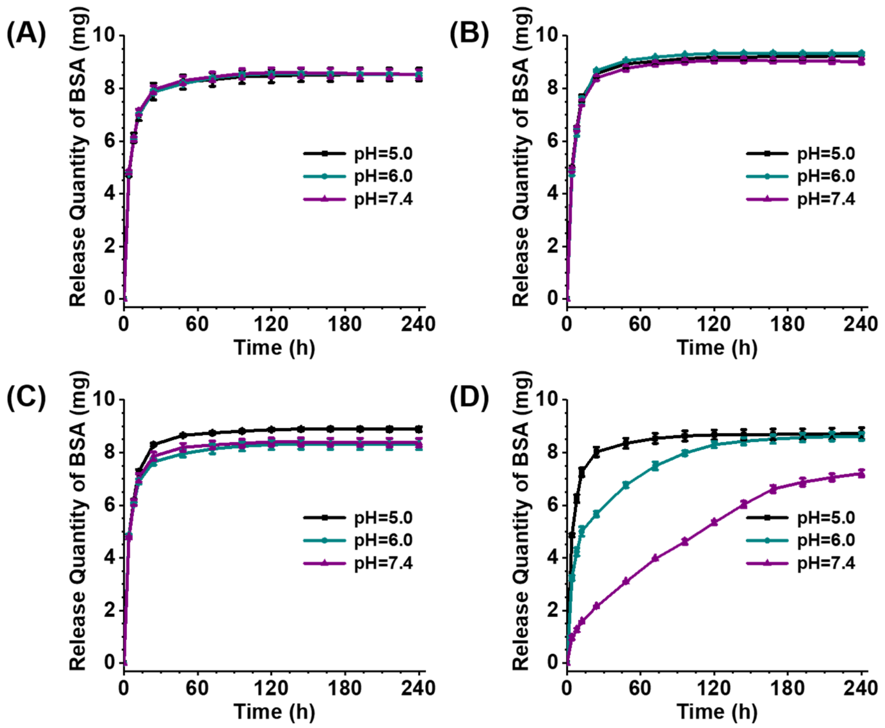

Figure S9. The release of BSA from (A) Ti-NTs, (B) Ti-NTs-D, (C) Ti-NTs-PCtrl and (D) Ti-NTs-P at different pH values. See Section *Loading and releasing of BSA* for details of the assay. Error bars denote the standard deviations over triplicate measurements with separate implants.

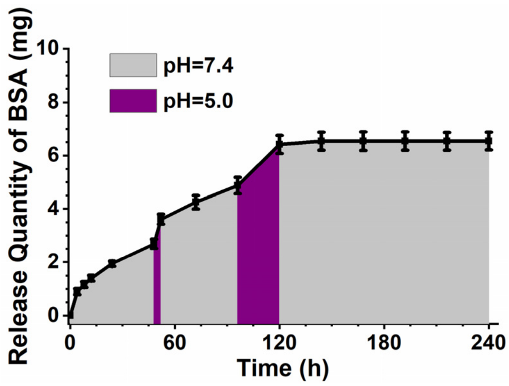

Figure S10. The release of BSA from Ti-NTs-P with a change in pH values at two randomly chosen time points (48 h and 96 h). See Section *Loading and releasing of BSA* for details of the assay. Error bars denote the standard deviations over triplicate measurements with separate implants.

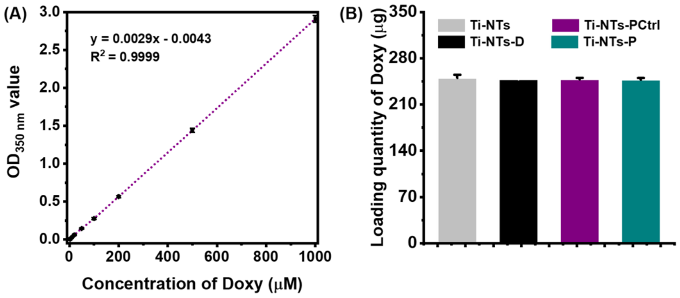

Figure S11. (A) The fitting curve of OD_{350} versus the concentrations of Doxy. (B) The quantity of Doxy loaded in the indicated substrates. See Section *Loading and releasing of doxycycline hyclate (Doxy)* for details of the assay. Error bars denote the standard deviations over triplicate measurements with separate implants.

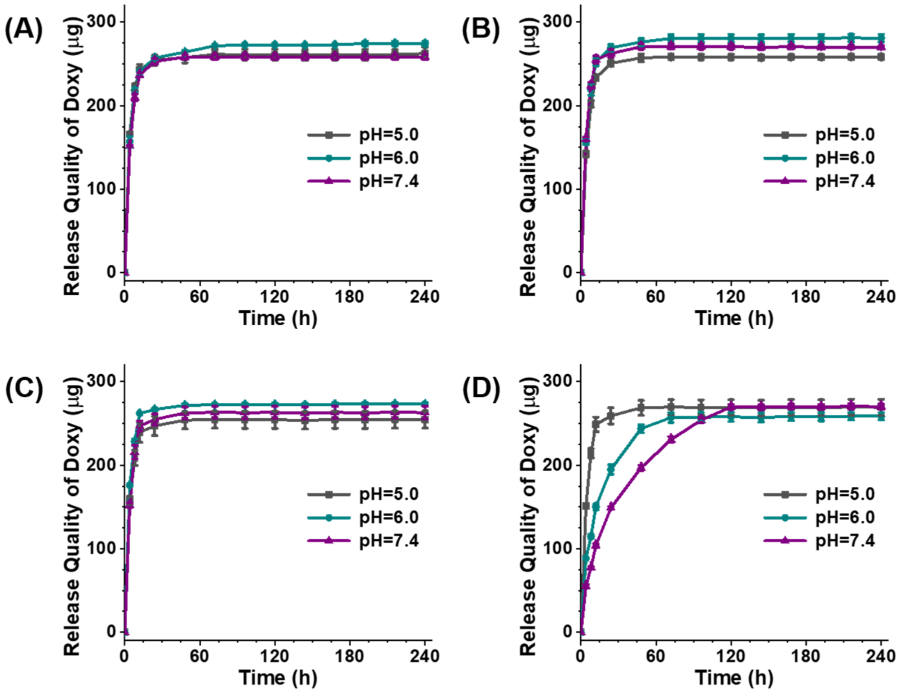

Figure S12. The release of Doxy from (A) Ti-NTs, (B) Ti-NTs-D, (C) Ti-NTs-PCtrl and (D) Ti-NTs-P at different pH values. See Section *Loading and releasing of doxycycline hyclate (Doxy)* for details of the assay. All error bars denote the standard deviations over triplicate measurements with separate implants.

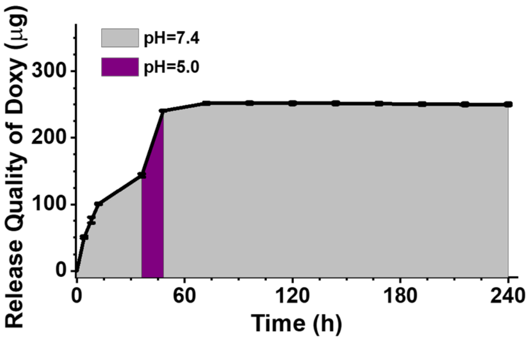

Figure S13. The release of Doxy from Ti-NTs-P with a change in pH values at two randomly chosen time points (36 h). See Section *Loading and releasing of Doxy* for details of the assay. Error bars denote the standard deviations over triplicate measurements with separate implants.

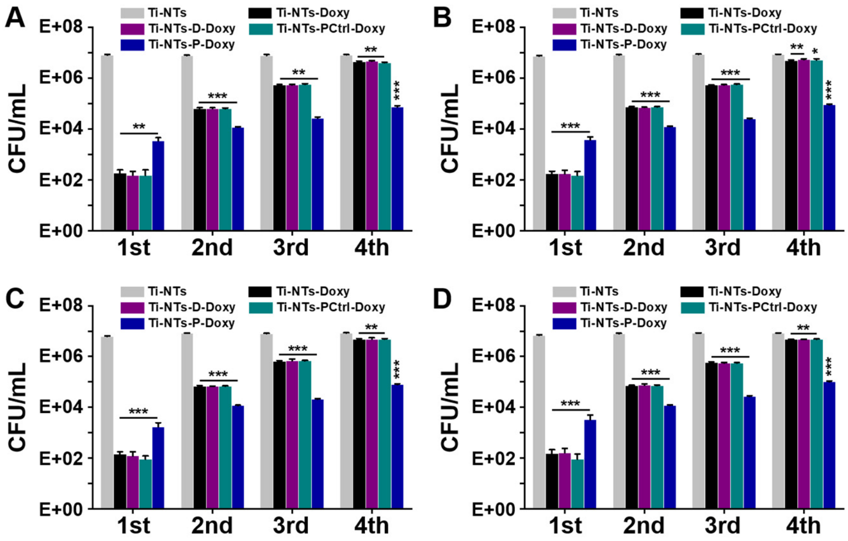

Figure S14. Bactericidal activity of the indicated substrates against (A) *S. aureus*, (B) *E. coli*, (C) *P. aeruginosa* and (D) *MRSA* in 1 h for four cycles. See Section *In vitro bactericidal assay of the surface with Doxy* for details of the assay. Error bars denote the standard deviations over triplicate measurements with separate implants.

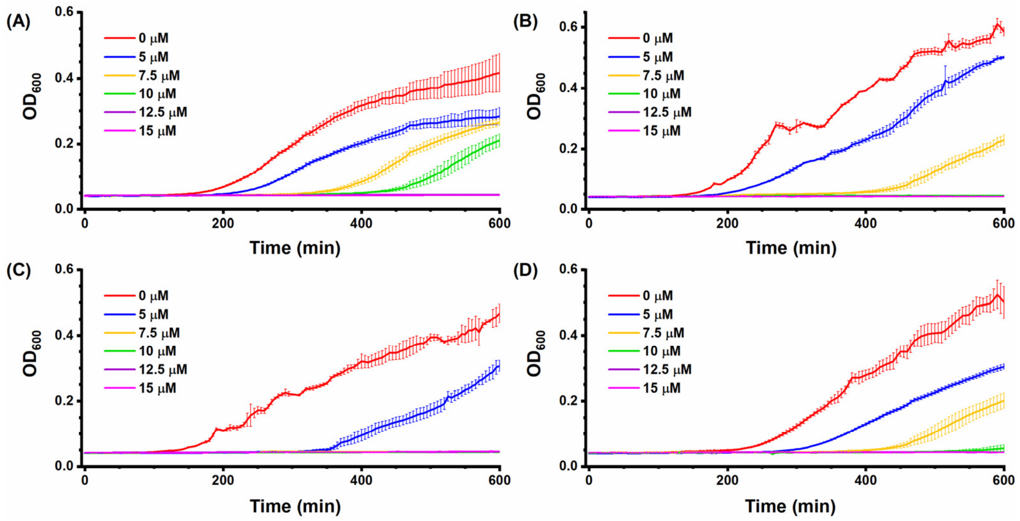

Figure S15. Bacterial growth curves of (A) *S. aureus*, (B) *E. coli*, (C) *P. aeruginosa*, and (D) *MRSA* under different concentrations of HHC36 peptides. See Section *Minimum inhibitory concentration (MIC) and minimum bactericidal concentration (MBC) assay* for details of the assay. All error bars denote the standard deviations over quadruplicate measurements with separate implants.

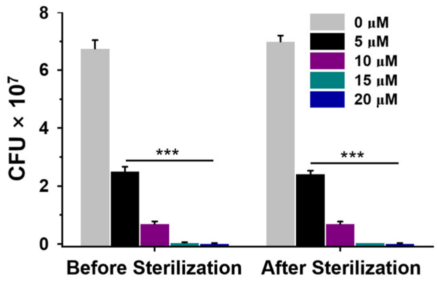

Figure S16. Bactericidal activity of HHC36 peptides against *S. aureus* before/after steam sterilization. *** denotes $p < 0.001$. See Section *In vitro bactericidal assay* for details of the assay. All error bars denote the standard deviations over triplicate measurements with separate implants.

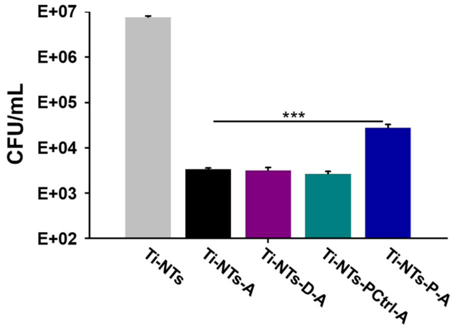

Figure S17. Bactericidal activity of the indicated substrates against *S. aureus* after steam sterilization. *** denotes $p < 0.001$. See Section *In vitro bactericidal assay* for details of the assay. All error bars denote the standard deviations over triplicate measurements with separately implants.

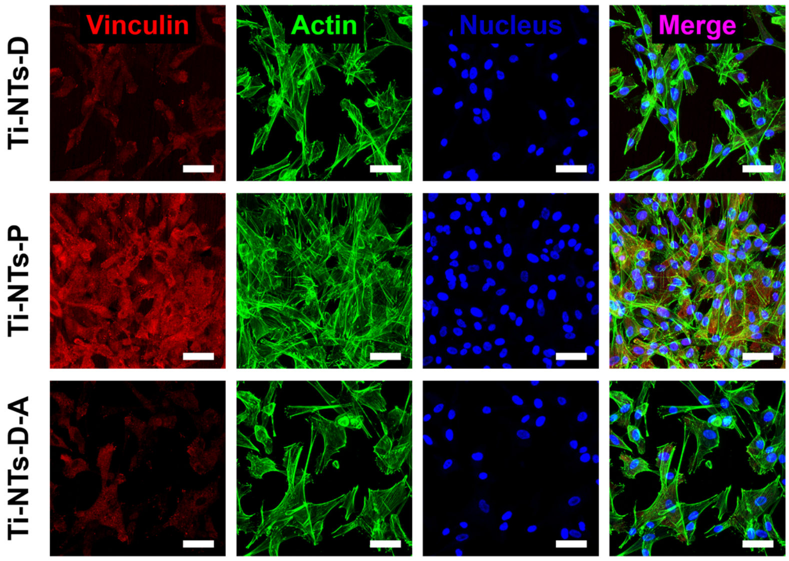

Figure S18. Confocal fluorescence microscopy images of *hBMSCs* on the indicated substrates. The cells were stained with vinculin, F-actin and DAPI after being cultured for 24 h. Scale bar, 50 μm. See Section *Immunofluorescence staining* for details of the assay.

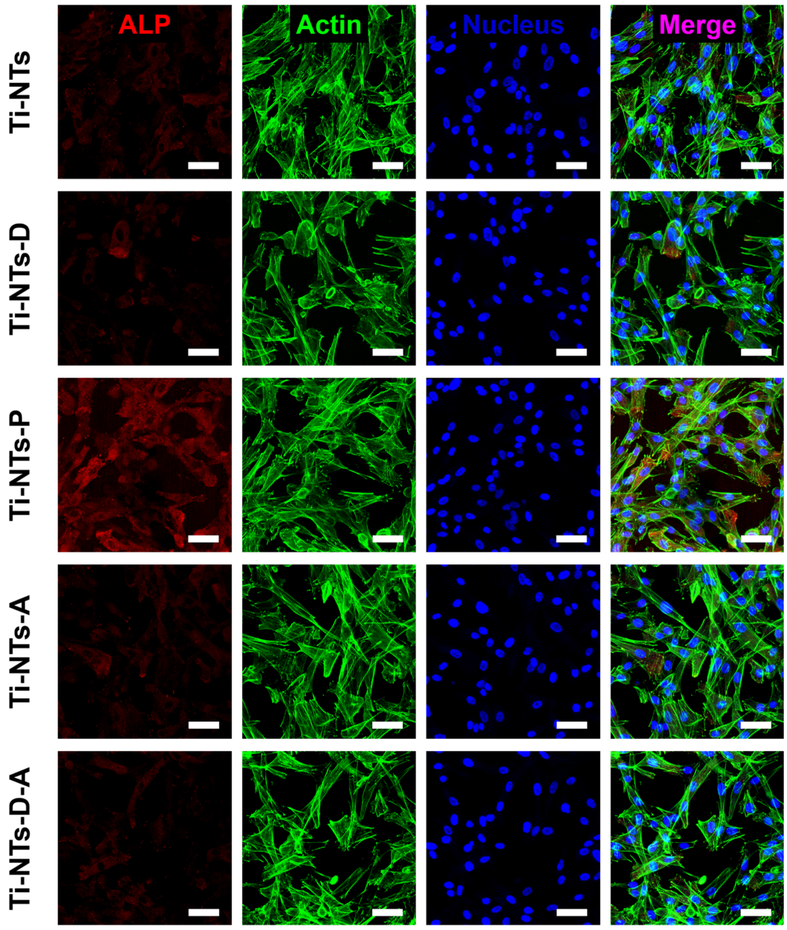

Figure S19. Immunofluorescence staining (ALP) of *hBMSCs* cultured on the indicated substrates for 7 days. The images were obtained by confocal fluorescence microscopy. Scale bar, 50 μm. See Section *Immunofluorescence staining* for details of the assay.

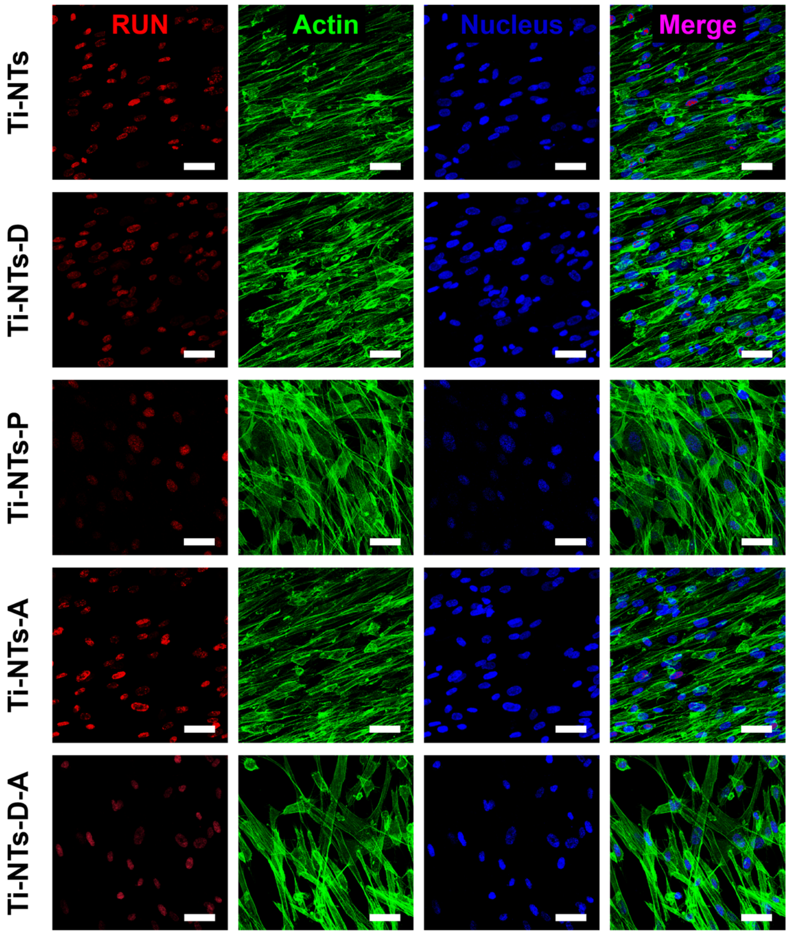

Figure S20. Immunofluorescence staining (RUNX-2) of *hBMSCs* cultured on the indicated substrates for 7 days. The images were obtained by confocal fluorescence microscopy. Scale bar, 50 μm. See Section *Immunofluorescence staining* for details of the assay.

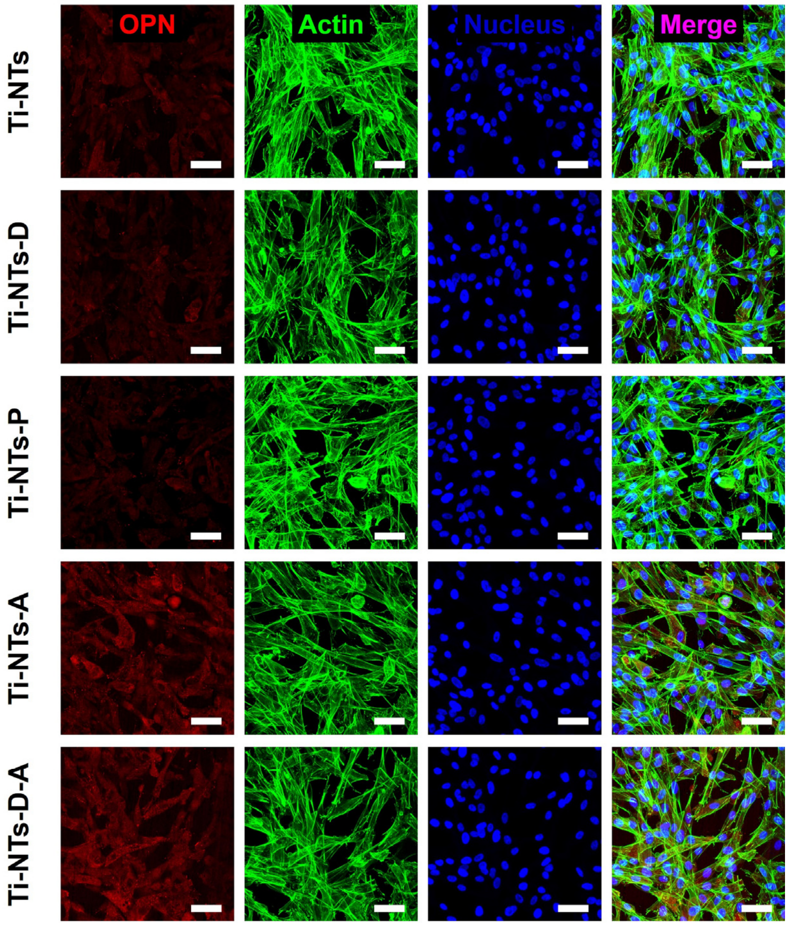

Figure S21. Immunofluorescence staining (OPN) of *hBMSCs* cultured on the indicated substrates for 14 days. The images were obtained by confocal fluorescence microscopy. Scale bar, 50 μm. See Section *Immunofluorescence staining* for details of the assay.

Table S1. The elemental compositions of the indicated surfaces determined by XPS. See Section *Surface characterization* for details of the assay.

Substrates	Composition (at. %)				N/Ti (%)	N/C (%)
	Ti 2p	C 1s	O 1s	N 1s		
**Ti-NTs**	28.1	16.8	53.8	1.3	4.6	7.7
**Ti-NTs-D**	7.8	56.9	28.2	7.1	91.0	12.4
**Ti-NTs-PCtrl**	26.4	18.6	53.3	1.7	6.4	9.1
**Ti-NTs-P**	12.4	37.5	49.6	0.5	4.0	1.3

Table S2. Primer set sequences for qRT-PCR. See Section *Quantitative reverse transcription polymerase chain reaction (qRT-PCR)* for details of the assay.

Gene	Forward primer	Reverse primer
GAPDH	5`-GCACCGTCAAGGCTGAGAAC-3`	5`-TGGTGAAGACGCCAGTGGA-3`
ALP	5`-CATGCTGAGTGACACAGACAAGAA-3`	5`-ACAGCAGACTGCGCCTGGTA-3`
RUNX2	5`-CACTGGCGCTGCAACAAGA-3`	5`-CATTCCGGAGCTCAGCAGAATAA-3`
COL-1	5`-GCTTGGTCCACTTGCTTGAAGA-3`	5`-GAGCATTGCCTTTGATTGCTG-3`
OPN	5`-GATGAATCTGATGAACTGGTCACTG-3`	5`-GGTGATGTCCTCGTCTGTAGCA-3`

Table S3 The abbreviations of the samples with doxycycline hyclate (Doxy).

Sample abbreviation	Treatment method
Ti-NTs-Doxy	Ti-NTs loaded Doxy
Ti-NTs-D-Doxy	Ti-NTs-D loaded Doxy
Ti-NTs-PCtrl-Doxy	Ti-NTs-PCtrl loaded Doxy
Ti-NTs-P-Doxy	Ti-NTs-P loaded Doxy
